# Molecular Characterization of Carbapenem Resistant *Klebsiella pneumoniae* and *Klebsiella quasipneumoniae* Isolated from Lebanon

**DOI:** 10.1038/s41598-018-36554-2

**Published:** 2019-01-24

**Authors:** Harout Arabaghian, Tamara Salloum, Sahar Alousi, Balig Panossian, George F. Araj, Sima Tokajian

**Affiliations:** 1 Department of Natural Sciences, School of Arts and Sciences, Lebanese American University, Byblos, 1401 Lebanon; 2 Department of Pathology and Laboratory Medicine, Faculty of Medicine, American University of Beirut Medical Center, Beirut, 1107 Lebanon

## Abstract

*Klebsiella pneumoniae* is a Gram-negative organism and a major public health threat. In this study, we used whole-genome sequences to characterize 32 carbapenem-resistant *K. pneumoniae* (CRKP) and two carbapenem-resistant *K. quasipneumoniae* (CRKQ). Antimicrobial resistance was assessed using disk diffusion and E-test, while virulence was assessed *in silico*. The capsule type was determined by sequencing the *wzi* gene. The plasmid diversity was assessed by PCR-based replicon typing to detect the plasmid incompatibility (Inc) groups. The genetic relatedness was determined by multilocus sequence typing, pan-genome, and recombination analysis. All of the isolates were resistant to ertapenem together with imipenem and/or meropenem. Phenotypic resistance was due to *bla*_OXA-48,_ *bla*_NDM-1_, *bla*_NDM-7,_ or the coupling of ESBLs and outer membrane porin modifications. This is the first comprehensive study reporting on the WGS of CRKP and the first detection of CRKQ in the region. The presence and dissemination of CRKP and CRKQ, with some additionally having characteristics of hypervirulent clones such as the hypermucoviscous phenotype and the capsular type K2, are particularly concerning. Additionally, mining the completely sequenced *K. pneumoniae* genomes revealed the key roles of mobile genetic elements in the spread of antibiotic resistance and in understanding the epidemiology of these clinically significant pathogens.

## Introduction

*Klebsiella pneumoniae*, a non-motile Gram-negative rod, is one of the leading bacterial pathogens causing hospital-associated infections through highly resistant clones, commonly referred to as classic *K. pneumoniae* (cKP)^1,2^_._ *K. pneumoniae* infections associated with a variety of circulating clones account for up to 8% of all nosocomial bacterial infections affecting neonates, the elderly, and the immunocompromised with mortality rates reaching as high as 40%^1^. Moreover, a rapidly spreading clinical pathotype, first described two centuries ago and designated as the hypervirulent *K. pneumoniae* (hvKP), is linked to community-acquired infections in healthy young individuals^3^. hvKP is in general characterized phenotypically by hypermucoviscous colonies linked to capsule overproduction and genotypically by specific capsular types and marker iron-acquisition molecules^3,4^.

Until 2001, carbapenemases were considered as the last resort drugs for the treatment of multi-drug resistant (MDR) *K. pneumoniae*^5^. This was before the first description of *K. pneumoniae* carbapenemase (KPC) production in isolates from North Carolina^5^. Since then, carbapenem-resistant *K. pneumoniae* (CRKP) have been frequently identified in multiple nosocomial settings throughout the world^6–8^. CRKP organisms are resistant to all β-lactams and often to other important therapeutic agents^9^. The most frequently encountered enzymes are the Ambler class A KPCs, class B metallo-β-lactamases (VIM, IMP, and NDM-1), and the class D OXA-type enzymes (OXA-48-like)^6^. Additionally, the upregulation of efflux pumps or loss of porins and the upregulation of ESBLs or AmpC β-lactamases can lead to carbapenem resistance^6^.

The incompatibility (Inc) group FIIK is the most common plasmid replicon found in *K. pneumoniae*^10^. Other common plasmid replicons include the IncR and IncL/M^11^. Numerous antimicrobial resistance genes (ARGs) co-exist on the same plasmid and are transferred together, leading to treatment failure in cases of severe infections^11,12^.

In this study, we conducted an in-depth molecular characterization of 24 MDR, eight extensively-drug resistant (XDR) *K. pneumoniae*, and two MDR *K. quasipneumoniae* clinical isolates. These included all of the CRKP isolates subsampled from a larger dataset of *K. pneumoniae* collected from 2012 to 2015 during routine clinical microbiology screening in a 350-bed tertiary care hospital in Lebanon. We used whole-genome sequences (WGS) to investigate and compare their genetic relatedness, antimicrobial resistance, and the presence of virulence determinants. The genomes were used to build a genomic landscape and were placed into a comprehensive context through a comparison with isolates recovered from various clinical sources worldwide. The outcome of this study also provided new insights into the pathogenesis and resistance of the previously under-recognized human pathogen *K. quasipneumoniae* in Lebanon.

## Results

### Isolates characteristics

Isolates were subsampled from a pool of *K. pneumoniae* collected between 2012–2015 as part of routine clinical microbiology screening of patients admitted to the American University of Beirut Medical Center (AUBMC). The inclusion criterion was non-susceptibility, defined as resistant or intermediate phenotypes to at least one of the three clinically tested carbapenems, namely imipenem, ertapenem, or meropenem following the Clinical and Laboratory Standards Institute (CLSI) guidelines^13^. All of the isolates were non-susceptible to ertapenem, 85.3% (29/34) to imipenem, and 70.6% (24/34) to meropenem (Supplementary Table S1a,b).

Resistance to meropenem was observed only when the isolate was simultaneously resistant to the remaining two carbapenems. In all cases where an isolate was resistant to only two carbapenems, these were ertapenem and imipenem. Regarding colistin, a last resort antibiotic used in the treatment of CRKP, 32.3% (11/34) of the isolates showed intermediate resistance according to the cut-off values proposed by Galani *et al*. (2008)^14^. 23% (8/34) of the isolates which were non-susceptible to at least one agent in all but two or fewer antimicrobial classes were classified as XDR (Supplementary Table S2)^15^.

**Figure 1:**
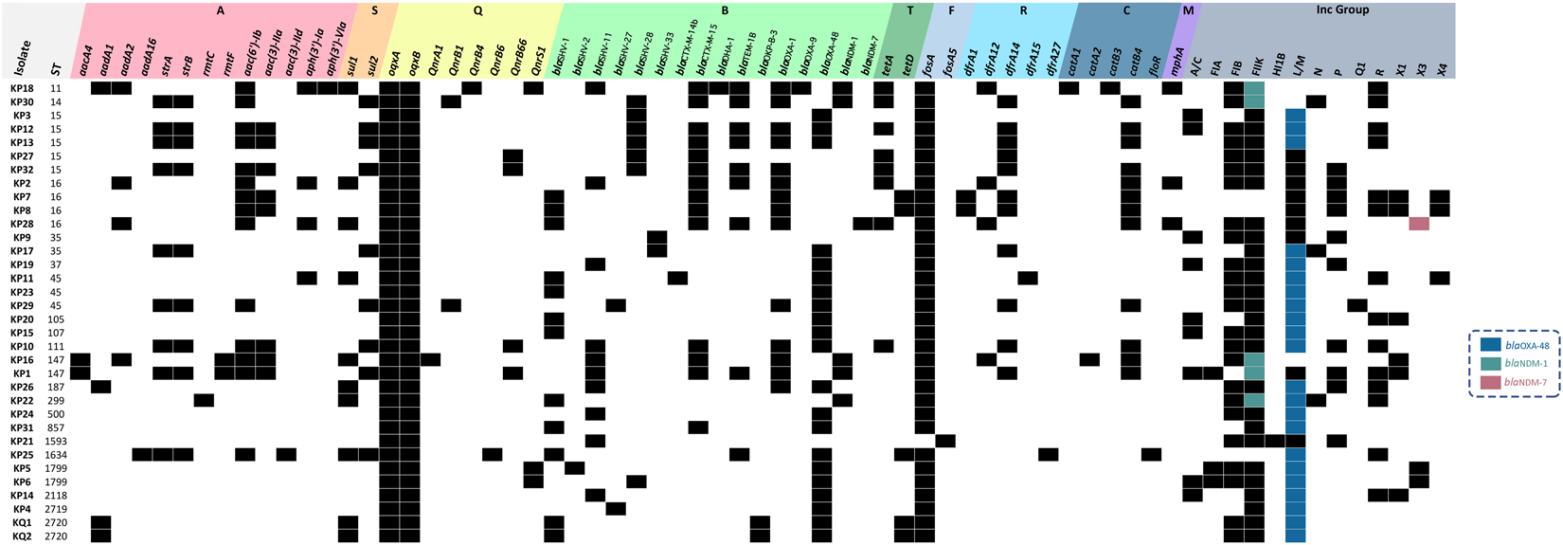
Sequence types, antibiotic resistance genes, and Inc groups detected by PCR and *in silico*. ST, Sequence type. Classes of antibiotic resistance genes are marked as follows: A, aminoglycoside resistance genes; S, sulfonamide resistance genes; Q, quinolone resistance genes; B, β-lactam resistance genes; T, tetracycline resistance genes; F, fosfomycin resistance genes; R, trimethoprim resistance genes; C, chloramphenicol resistance genes; M, macrolide resistance genes; Inc Groups, plasmid incompatibility groups.

PCR based ARG detection and ResFinder applied to the WGS revealed the presence of 54 distinct genes, each conferring resistance to one of the nine antibacterial classes: aminoglycosides, sulfonamides, quinolones, β-lactams, tetracyclines, fosfomycin, trimethoprim, chloramphenicol, and macrolides (Fig. 1). In instances where a carbapenemase gene was absent, core chromosomal genes involved in carbapenem resistance, such as genes encoding for outer membrane porin proteins OmpK35 and OmpK36, were analyzed. Carbapenem resistance patterns detected through antimicrobial susceptibility testing were in agreement either with the detection of various carbapenemase genes or with modified chromosomal genes linked to carbapenem resistance.

*In silico* whole-genome and PCR-based analysis were used to assess the prevalence of ARGs. *bla*_OXA-48_ (61.2%; 21/34) was the most common carbapenemase detected, followed by *bla*_NDM-1_ (14.7%; 5/34) and *bla*_NDM-7_ (2.9%; 1/34). *bla*_CTX-M-15_ (41.2%; 14/34) was the most common ESBL variant detected. *bla*_OKP-B-3_, a chromosomally-encoded β-lactamase, was detected in the *K. quasipneumoniae* isolates. Carbapenemase-encoding genes were absent in seven isolates (KP2, KP7–9, KP21, KP27, and KP32) showing phenotypic resistance to carbapenems associated with the presence of an ESBL coupled with porin loss or modification^6^. KP2, KP7–9 all carried multiple β-lactamases and mutated versions of the outer membrane porins OmpK35 and OmpK36 with at least seven and 13 mutations found in each, respectively. KP21 carried a *bla*_SHV-11_ ESBL and a truncated OmpK36 porin due to a premature stop codon in the *ompK36* gene. This variation was also observed in KP27 and KP32. Since premature stop codons were only detected in *ompK36*, its non-synonymous changes within all isolates were assessed in an attempt to detect positive selection exerted on these mutations. Hence, SNAP (http://www.hiv.lanl.gov) was used to calculate synonymous and non-synonymous substitution rates based on the codon-aligned nucleotide sequences of an intact *ompK36* sequence (accession # KY086538.1) against two other references (accession # KY086537.1 and MG577056.1) obtained from NCBI and the 34 *ompK36* sequences retrieved from the isolates’ WGS. Upon performing the analysis using SNAP, the number of observed and potential synonymous and non-synonymous substitutions were calculated and a plot was generated (Supplementary Fig. S1). These were then used to calculate the ratio of non-synonymous to synonymous substitutions (dN/dS)which was equal to 1.2589.

### Plasmid studies

PCR-based replicon typing (PBRT) was performed and PlasmidFinder was applied to the WGS to successfully identify 13 Inc groups (Fig. 1). Using these two complementary methods, groups that were not covered by the PBRT kit, such as IncX4, were detected by PlasmidFinder, while many other missed plasmids through *in silico* analysis of draft genomes were confirmed by PBRT.

At least three different Inc groups were identified in the majority of the tested isolates (91.2%; 31/34) (Fig. 1). The highest number of Inc groups (n = 7) was detected in KP1. The PLAsmid Constellation NETwork (PLACNETw) was used to reconstruct the plasmids from paired-end reads and it showed the presence of one big multi-replicon plasmid, carrying IncFIIK, IncFIA, and IncR, and three relatively smaller plasmids having IncA/C, IncL/M, and IncX1 replicons (Supplementary Fig. S2). The IncP replicon was the only one missing from the short reads.

IncFIIK and IncL/M were the most common, each present in 91.2% (31/34) of the isolates, followed by IncFIB (70.1%; 24/34), IncR (41.2%; 14/34), IncP (29.4%; 10/34), IncA/C (26.5%; 9/34), and IncX1 (17.6%; 6/34) (Fig. 1).

**Figure 2:**
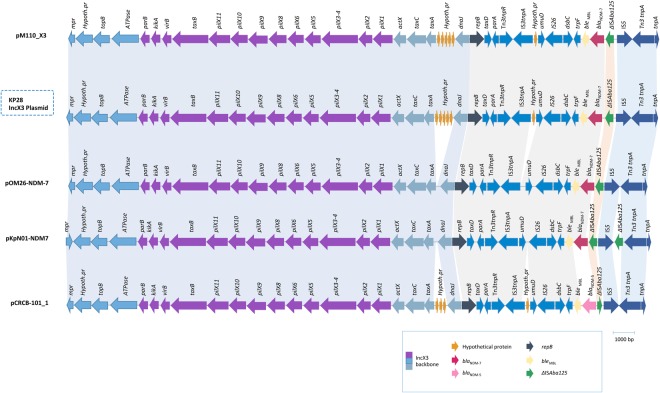
Comparison of the genetic environment of *bla*_NDM-7_ allocated on an IncX3 plasmid in KP28. *bla*_NDM-7_ was carried by an IS*5-*$\triangle$IS*Aba125-bla*_NDM-7_*-ble*_MBL_*-trpF-dsbC*-IS*26* genetic element.

The IncX group of plasmids is relatively poorly studied compared to other Inc types^16^. The IncX4 plasmid, which carried a *bla*_CTX-M-14b_ gene and detected for the first time in Lebanon, was only found in KP11. KP28 was positive for an IncX3 plasmid, and was the only isolate positive for *bla*_NDM-7._ To our knowledge, this is the first report of IncX3 carrying *bla*_NDM-7_ in Lebanon. Upon retrieving the complete plasmid sequence from the KP28 genome and BLASTing against GenBank, the IncX3 plasmid was found to be highly identical to previously published plasmids with different NDM subtypes. For instance, it was 99% similar to *bla*_NDM-7_ harboring pKpN01-NDM7 from *K. pneumoniae* (CP012990.1) isolated in Canada; 99% similar to *bla*_NDM-7_ encoding pM110_X3 from *Escherichia coli* (accession # AP018141.1) isolated in Myanmar; 97% similar to *bla*_NDM-7_ harboring pOM26-1 from *E. coli* (accession # KP776609.1) in the Arabian Peninsula, and 99% similar to pCRCB-101_1 (accession # NZ_CP024820.1) encoding *bla*_NDM-5_ found in *Citrobacter freundii* isolated in South Korea. Analysis of the genetic environment of IncX3 reflected the presence of only one copy of $\triangle$IS*Aba125* in the KP28 plasmid (Fig. 2).

### Virulence analysis

**Figure 3:**
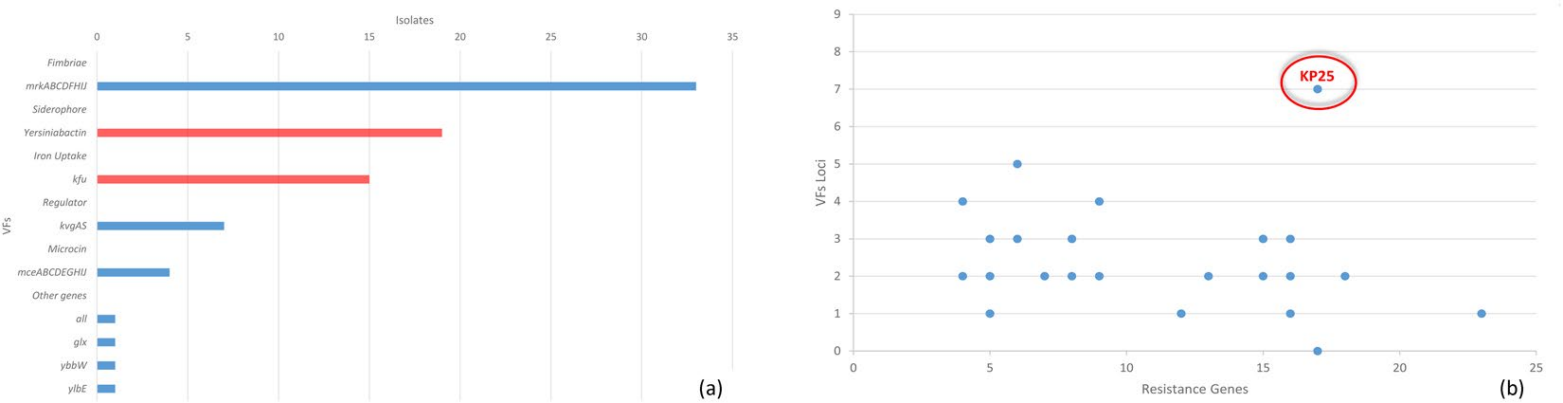
Prevalence of different virulence factors detected and the total number of virulence factors in a relationship with the total number of resistance genes. (**a**) Frequency of important VFs detected among the isolates. (**b**) Blue dots representing one or more isolate with a specific number of VFs and ARGs. Encircled is an emerging type of *K. pneumoniae* with a high number of both VFs and ARGs.

Virulence profiling of WGS data revealed that fimbriae encoding genes, *mrkABCDFHIJ*, were the most common being detected in 94.1% (32/34) of the isolates. The yersiniabactin siderophore encoded by the yersiniabactin locus (*ybtS, ybtX, ybtQ, ybtP, ybtA, irp2, irp1, ybtU, ybtT, ybtE, fyuA*) was detected in 55.9% (19/34) of the isolates, while the *kfu* iron uptake system was found in 35% (12/34) of the isolates. KP25 was the only isolate positive for *all* (the activator of the allantoin regulon), *glx* (*glxK* and *glxR* encoding for enzymes in the glycerate pathway, *ybb* (encoding an allantoin permease) and *ylbE* (a putative cytoplasmic protein) (Fig. 3a). The highest number of virulence factors (VFs) were detected in KP6 (Supplementary Table S3). Isolates were tested for hypermucoviscosity, one of the markers of hypervirulence by performing the string test. All of the isolates were negative with the exception of KP32 (Supplementary Table S3).

Isolates identified as *K. quasipneumoniae* solely harbored the fimbriae encoding locus *mrkABCDFHIJ*.

The integrative and conjugative element, ICE*Kp*, which mobilizes the yersiniabactin locus was further analyzed following the classification suggested by Lam *et al*.^17^. Four ICE*Kp* types (ICE*Kp3–5* and ICE*Kp12*) carrying the yersiniabactin siderophore locus were detected among the isolates (Supplementary Table S4). In KP5 (from a deep throat aspirate; 2013), KP6 (from a cyst; 2013), and KP21 (from an incision site; 2014), the yersiniabactin siderophore locus was detected on an IncFIB type of plasmid similar to INF167_p0001 (accession # KY454639.1). INF167_p0001 was detected in *K. pneumoniae* INF167 isolated from a urine sample in Australia in 2013^17^. Plasmids harbored by KP5 and KP6 were identical and highly similar to INF167_p001; three IS*Ec63* were missing from KP5 and KP6 compared to INF167_p001, while all the three IS*Ec63* were present in KP21.

Assessing the convergence of VFs and ARGs revealed that KP25 had genes encoding for seven VFs and 15 ARGs (Figs 1 and 3b) (Supplementary Table S3).

### Isolates typing

**Figure 4:**
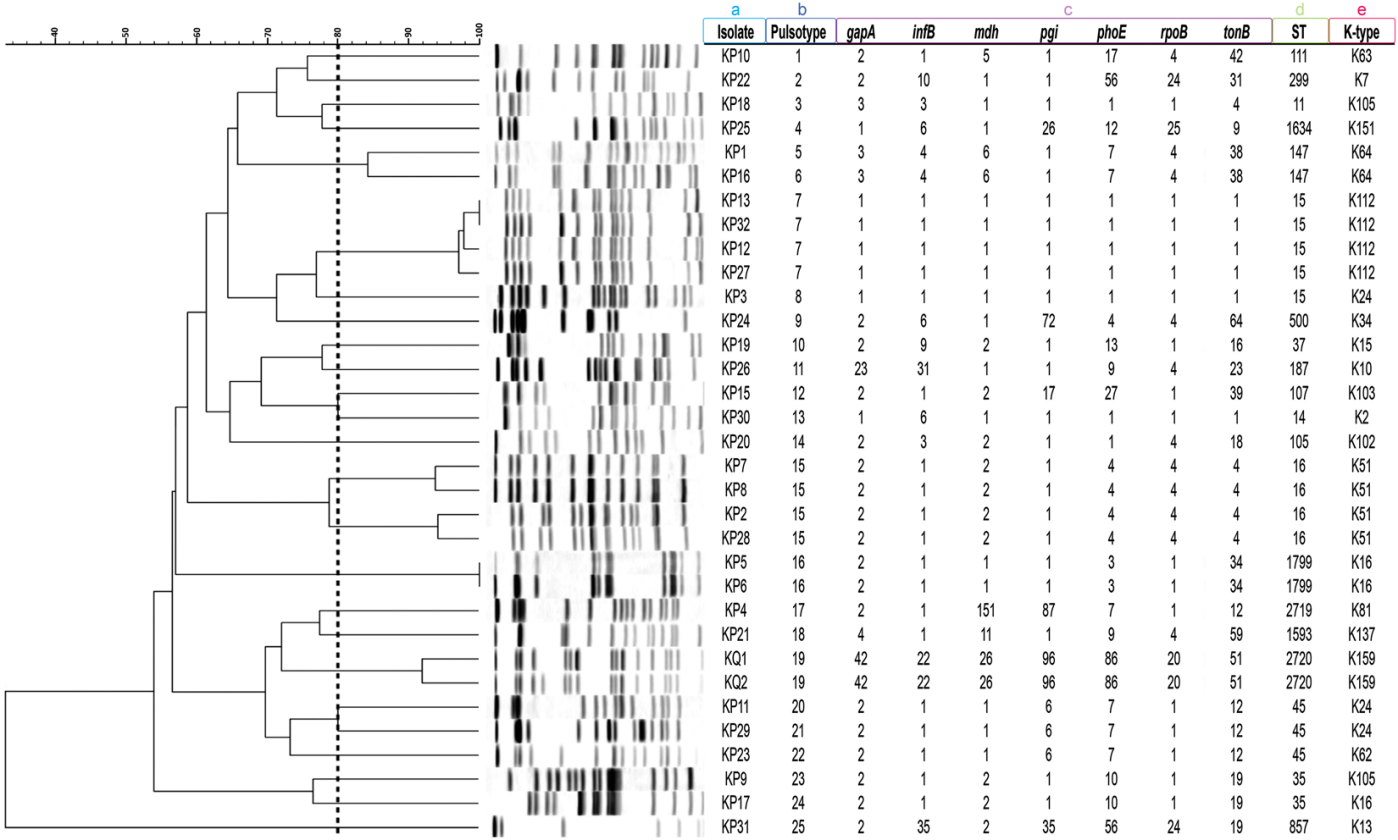
PFGE profiles, pulsotypes, seven-gene MLST profiles, ST, and K-type of the sequenced isolates. Dendogram generated by BioNumerics software version 7.6.1 showing the relationship of the isolates based on their banding patterns generated by XbaI restriction digestion. a, isolate name; b, pulsotype number; c, seven MLST housekeeping genes profile; d, sequence type; e, K-type.

In total, 21 different sequence types (STs) were detected: ST15 (14.7%; 5/34), ST16 (11.8%; 4/34), ST45 (8.8%; 3/34), ST35 (5.9%; 2/34), ST147 (5.9%; 2/34), ST1799 (5.9%; 2/34), ST2720 (5.9%; 2/34), and 14 singletons (41.2%; 14/34). *In silico* and PCR-based multilocus sequence typing (MLST) results were in accordance. PCR-based results were used to assign novel STs (ST2719 and ST2720). It is noteworthy that although pulsotypes cannot be used to confirm high genetic relatedness of strains, pulsed-field gel electrophoresis (PFGE) was performed to only screen for the presence of an outbreak scenario during 2012–2015 in the hospital. A total of 25 different pulsotypes were identified (Fig. 4). The two *K. quasipneumoniae* clustered along with seven other *K. pneumoniae*. Isolates with identical STs, single or double locus variants, clustered together, except for KP30 (ST14), a single locus variant (SLV) of ST15 that clustered instead with KP15 (ST107). It is noteworthy that KP31 had a distinct PFGE pattern from all the other isolates, while KP14 was untypable and did not give any bands, even after using a secondary enzyme, AvrII.

The *wzi* capsular gene was sequenced to assign a capsular type (K-type) to individual isolates. Based on that, thirteen different K-types were identified. 14.7% (5/34) of the isolates were typed as K15/K17/K50/K51/K52, while 26.5% (9/34) were not linked to any K-type. Due to the low resolution provided by sequencing a single *wzi* gene, the complete *cps* locus was targeted instead based on the WGS data using Kaptive. This resulted in the identification of 20 different K-types which were subsequently adopted throughout the manuscript^18^. No significant correlation was found upon comparing the STs, K-types, β-lactamases, incompatibility groups, and virulence features, highlighting the level of diversity among the isolates (Table 1).

**Table 1:** Molecular characterization of all STs detected.

ST	K-type	Inc groups	β-lactam resistance genes	Virulence factors
ST15 (n = 5)	K24 (n = 1), K112 (n = 4)	A/C (n = 2), FIB (n = 5), FIIK (n = 5), L/M (n = 5), P (n = 1), R (n = 2)	*bla*_SHV-28_ (n = 5), *bla*_CTX-M-15_ (n = 4), *bla*_TEM-1B_ (n = 3), *bla*_OXA-1_ (n = 3), *bla*_OXA-48_ (n = 3)	yersiniabactin (n = 5), *kfu* (n = 5), *mrkABCDFHIJ* (n = 5)
ST16 (n = 4)	K51 (n = 4)	FIB (n = 2), FIIK (n = 2), L/M (n = 4), P (n = 3), R (n = 2), X1 (n = 2), X3 (n = 1), X4 (n = 2)	*bla*_SHV-1_ (n = 3), *bla*_SHV-11_ (n = 1), *bla*_CTX-M-15_ (n = 4), *bla*_TEM-1B_ (n = 2), *bla*_OXA-1_ (n = 4), *bla*_NDM-7_ (n = 1)	yersiniabactin (n = 2), *mrkABCDFHIJ* (n = 4)
ST45 (n = 3)	K24 (n = 2), K62 (n = 1)	FIB (n = 3), FIIK (n = 3), Q1 (n = 1), L/M (n = 3), R (n = 1), X4 (n = 1)	*bla*_SHV-1_ (n = 2), *bla*_SHV-27_ (n = 1), *bla*_CTX-M-14b_ (n = 1), *bla*_OXA-1_ (n = 1), *bla*_OXA-48_ (n = 3)	yersiniabactin (n = 2), *mrkABCDFHIJ* (n = 3)
ST35 (n = 2)	K105 (n = 1), K12 (n = 1)	A/C (n = 1), FIB (n = 1), FIIK (n = 2), L/M (n = 2), N (n = 1), P (n = 1)	*bla*_CTX-M-14b_ (n = 2), *bla*_OXA-48_ (n = 1)	yersiniabactin (n = 1), *kfu* (n = 2), *mrkABCDFHIJ* (n = 2), *kvg* (n = 2)
ST147 (n = 2)	K64 (n = 2)	A/C (n = 1), FIA (n = 1), FIB (n = 1), FIIK (n = 2), L/M (n = 1), P (n = 1), R (n = 1), X1 (n = 2)	*bla*_SHV-11_ (n = 2), *bla*_CTX-M-15_ (n = 2), *bla*_TEM-1B_ (n = 1), *bla*_OXA-1_ (n = 2), *bla*_NDM-7_ (n = 2)	yersiniabactin (n = 1), *mrkABCDFHIJ* (n = 1)
ST1799 (n = 2)	K16 (n = 2)	A/C (n = 1), L/M (n = 2), FIA (n = 2), FIB (n = 2), FIIK (n = 2), X3 (n = 2)	*bla*_SHV-28_ (n = 1), *bla*_LEN12_ (n = 1), *bla*_OXA-48_ (n = 2)	yersiniabactin (n = 2), *kfu* (n = 2), *mrkABCDFHIJ* (n = 2), *mceABCDEGHIJ* (n = 2), *kvg* (n = 2)
ST2720 (n = 2)	K159 (n = 2)	L/M (n = 2), FIB (n = 2), FIIK (n = 2)	*bla*_SHV-1_ (n = 2), *bla*_OKP-B-3_ (n = 2), *bla*_OXA-48_ (n = 2)	*mrkABCDFHIJ* (n = 2), *kfu* (n = 2)
ST11 (n = 1)	K105	FIB, FIIK, R	*bla*_SHV-11_, *bla*_CTX-M-15_, *bla*_DHA-1_, *bla*_TEM-1B_, *bla*_OXA-1_, *bla*_OXA-9_, *bla*_NDM-1_	*mrkABCDFHIJ*
ST14 (n = 1)	K2	FIB, FIIK, N, R	*bla*_SHV-28_, *bla*_CTX-M-15_, *bla*_TEM-1B_, *bla*_OXA-1_, *bla*_NDM-1_	*mrkABCDFHIJ*
ST37 (n = 1)	K15	A/C, FIB, FIIK, L/M, P	*bla*_SHV-11_, *bla*_OXA-48_	yersiniabactin*, mrkABCDFHIJ*
ST105 (n = 1)	K102	A/C, FIIK, L/M, R, X1	*bla*_SHV-1_, *bla*_OXA-48_	yersiniabactin*, mrkABCDFHIJ, mceABCDEGHIJ*
ST107 (n = 1)	K103	A/C, FIB, FIIK, L/M,	*bla*_SHV-1_, *bla*_OXA-48_	yersiniabactin*, mrkABCDFHIJ*
ST111 (n = 1)	K63	FIB, FIIK, L/M, P, R	*bla*_SHV-11_, *bla*_CTX-M-15_, *bla*_OXA-1_, *bla*_OXA-48_	yersiniabactin*, mrkABCDFHIJ*
ST187 (n = 1)	K10	FIB, FIIK, L/M, P, R	*bla*_SHV-11_, *bla*_OXA-1_, *bla*_OXA-48_	yersiniabactin*, mrkABCDFHIJ*
ST299 (n = 1)	K7	FIB, FIIK, L/M, N, R	*bla*_SHV-1_, *bla*_NDM-1_	*mrkABCDFHIJ, kvg*
ST500 (n = 1)	K34	FIB, FIIK, L/M	*bla*_SHV-11_, *bla*_OXA-48_	*kfu, mrkABCDFHIJ, kvg*
ST857 (n = 1)	K13	FIIK, L/M	*bla*_SHV-11_, *bla*_CTX-M-15_, *bla*_OXA-48_	yersiniabactin*, kfu, mrkABCDFHIJ*
ST1593 (n = 1)	K137	HI1B, FIB, FIIK, L/M, P	*bla*_SHV-11_, *bla*_OXA-48_	yersiniabactin*, mrkABCDFHIJ*
ST1634 (n = 1)	K151	L/M, R	*bla*_SHV-1_,*bla*_TEM-1B_, *bla*_OXA-48_	*kfu, mrkABCDFHIJ, all, glx, ybb, ylbE, kvg*
ST2118 (n = 1)	K46	A/C, FIIK, L/M, R, X1	*bla*_SHV-11_, *bla*_OXA-48_	*mrkABCDFHIJ*
ST2719 (n = 1)	K81	FIIK, L/M	*bla*_SHV-27_, *bla*_OXA-48_	*mrkABCDFHIJ*

Twenty-one detected STs and their molecular features including K-types, Inc groups, β-lactamase genes, and selected virulence features.

### Genome Statistics and Comparative Analysis

**Figure 5:**
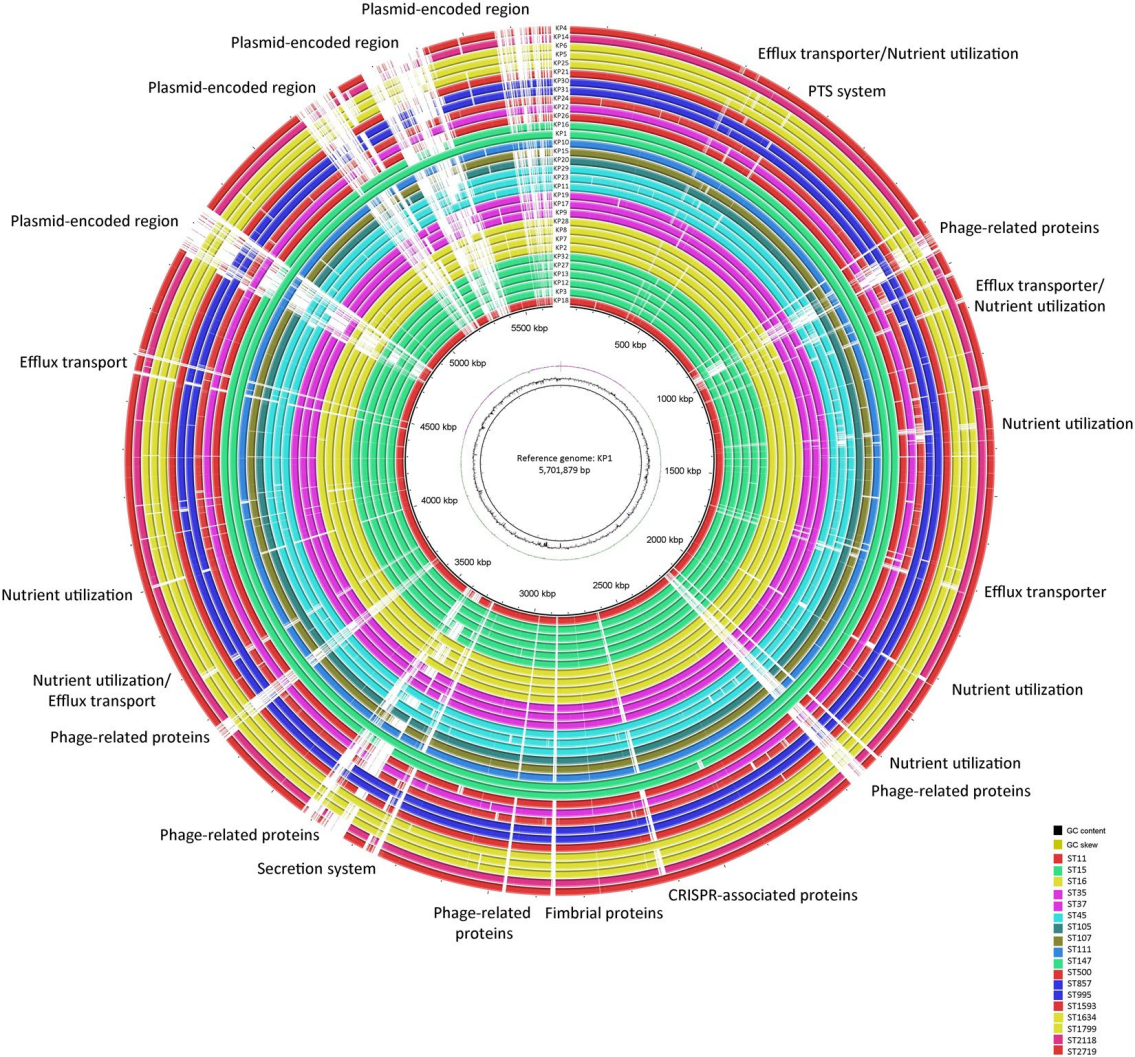
Circular genomic map and genome comparison of 32 *K. pneumoniae* genomes. The circle is divided into arcs representing the genomes as labeled. The black histogram represents the GC content and purple-green histogram represents the GC deviation. The isolates are colored according to their STs. The function of genes which were part of major deletion events are labelled at the edge of the rings. KP1 was used as a reference genome.

On average, the 34 genomes consisted of 5,516,119 ±134,003 bp in 92 ± 16.98 contigs and had an average GC content of 57% ± 0.22% with a 30x reads coverage (Supplementary Fig. S3). 594 ± 3.95 subsystems, 5,221 ± 178 coding sequences and 113 ± 7.22 RNAs were annotated on average by isolate (Supplementary Table S5). Comparative genome analysis using BRIG and KP1 (ST147) as the reference revealed deletion events involving membrane transport genes (e.g. ABC Transporter system, RND efflux system), fimbrial and type VI secretion system encoding genes. The type VI secretion system proteins were detected in six isolates. The circular genome based comparison may be overlooking key acquisition/loss events in the remaining strains that are not present in the used reference genome (Fig. 5). Differences in the phage content of the isolates were observed upon using PHASTER annotation tool. In total, 43 different phages were identified. The number of detected phages ranged from one in KP14 and KP29 to 11 phages in KP18 with an average of 4.27 ± 2.23 phages per isolate. *Salmonella* phage RE-2010 (accession # NC_019488) was the most common phage, being identified in 38.2% (n = 13) of the isolates (Supplementary Table S6).

**Figure 6:**
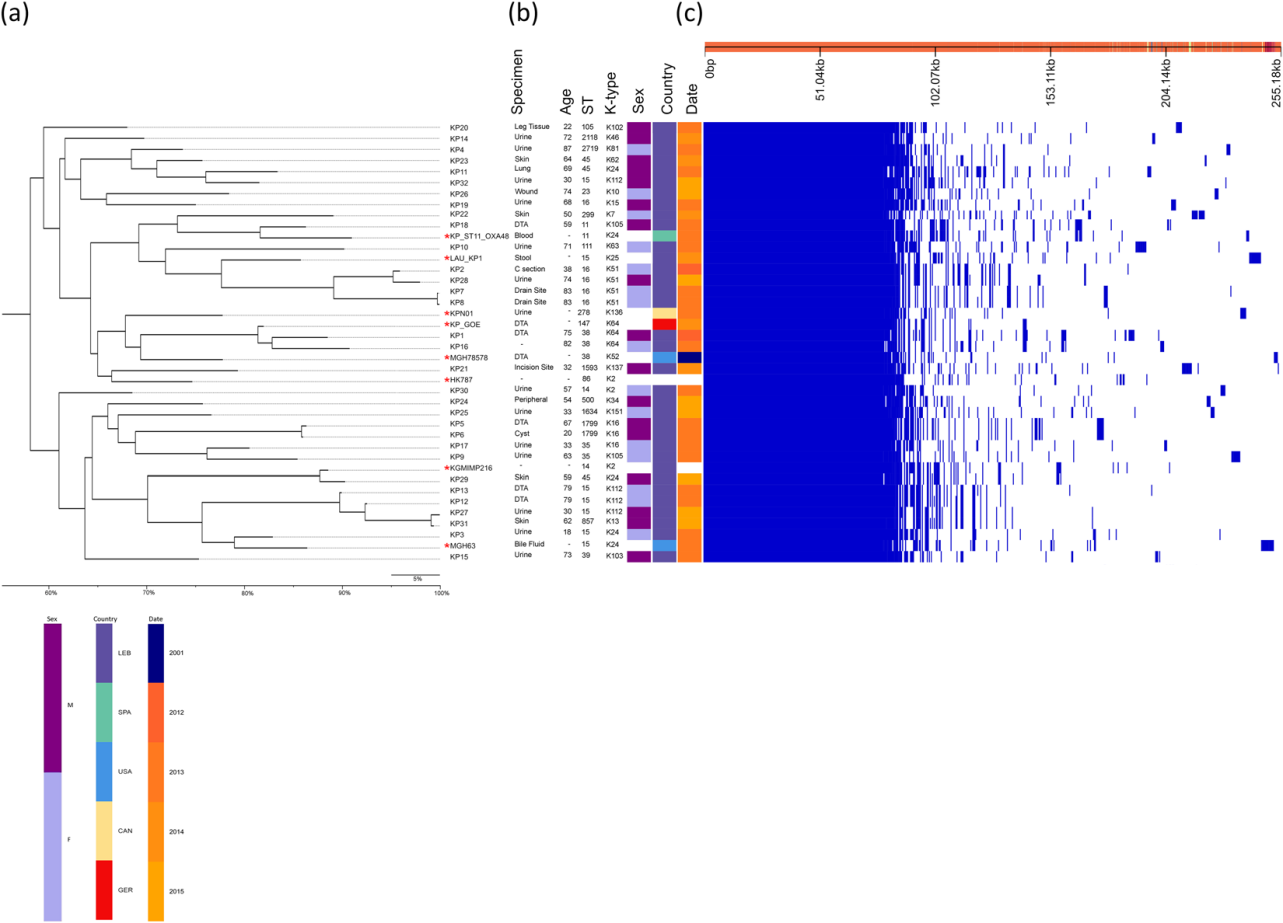
Pan-genome similarity matrix, patient and isolate data, and maximum likelihood tree based on core and accessory genomes. (**a**) Maximum likelihood phylogenetic tree based on the alignment of accessory genomes was generated using FastTree 2^61^. *indicates reference genomes. (**b**) Patient metadata including specimen type, age, ST, K-type, sex (M: male; F: female), country (LEB: Lebanon; SPA: Spain; USA: United States of America; CAN: Canada; GER: Germany), and collection date. (**c**) Pan-genome was constructed using Roary based on the core and accessory genes showing phylogenetic relatedness of the isolates by blue (present) and white (absent) fragments^60^.

Pan-genome analysis revealed a total of 11,375 genes which were separated according to their prevalence within the isolates according to different categories including core (genes shared by 99–100% of the isolates), soft (genes shared by 95–99% of the isolates), and accessory genomes (genes shared by less than 95% of the isolates) (Supplementary Fig. S4). 4,005 core and 7,214 unique accessory genes were identified. Isolates sharing the same accessory genes were clustered together (Fig. 6). All nodes had above 95% bootstrap support. A considerable correlation was not observed between sites of infection, age of patient, and overlap of resistance phenotypes. The phylogenetic analysis revealed close association between some of the ST-types (ST15, ST16, ST1799, and ST147) and their K-types, with the exception of one ST15 isolate (KP32) that clustered separately from the remaining ST15 isolates. This observed ST paraphyly is a direct implication of the robustness and value of whole-genome-based typing approaches over ST analysis, which is based only on seven housekeeping genes.

**Figure 7:**
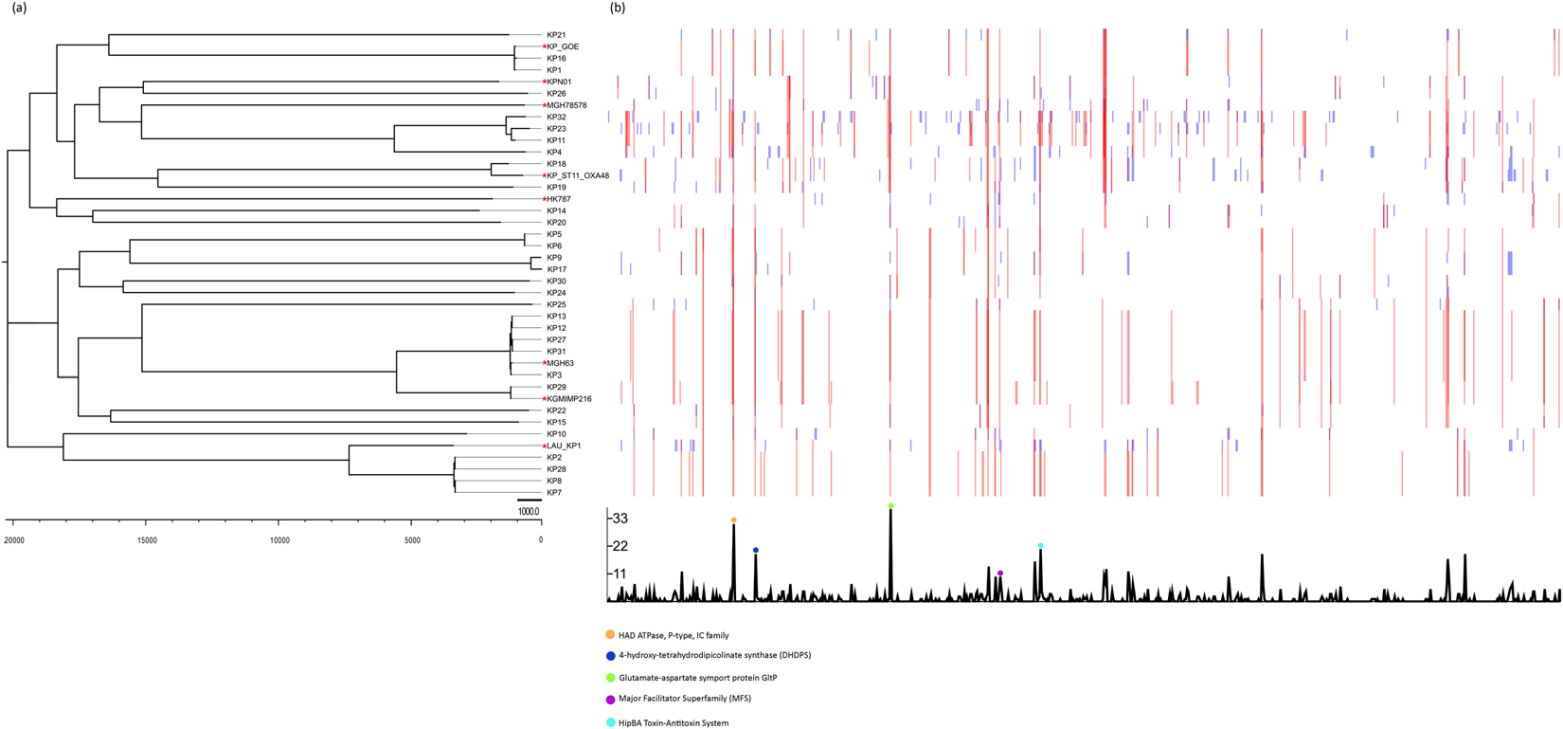
Core genome variation in recombination and SNPs. (**a**) Maximum likelihood phylogenetic tree based on core genome SNPs. *indicates reference genomes. (**b**) Recombination densities were detected between the samples using Gubbins^62^.

As an added layer of depth in the analysis, assessment of recombination events between the core genomes segregated the isolates based on predicted regional hotspots and unique single nucleotide polymorphisms (SNPs) denoted in Fig. 7 as red and blue vertical lines, respectively. 99,852 core genome SNPs, defined as base variations within the core genomes of the isolates and the eight reference sequences, were detected among the isolates and reference genomes and their alignment was used to generate a maximum-likelihood phylogenetic tree. This high resolution provided by WGS and SNP analysis has made such approaches the ultimate genotyping method currently available.

Using BLASTn search against the non-redundant nucleotide database we were able to associate the highly recombinant regions to known proteins such as: the P-type HAD ATPase IC family protein, 4-hydroxy-tetrahydrodipicolinate synthase, Glutamate/Aspartate Symporter, HipAB, and a protein of the Major Facilitator Superfamily.

## Discussion

In this study, we performed an in-depth molecular characterization of 24 MDR, eight XDR *K. pneumoniae*, and two MDR *K. quasipneumoniae* clinical isolates. Using a whole-genome-based approach, we compared the genetic relatedness, antimicrobial resistance, and the virulome of the isolates under study along with other international clones. Significant variability was observed in the resistance and plasmid Inc groups among the same and different ST and K-types. None of the ARGs could be associated with a specific Inc group, except for *bla*_OXA-48_, which was confined to IncL/M, and *bla*_NDM-1,_ to the IncF replicons. Several virulence determinants were detected, with the yersiniabactin siderphore locus being notably carried either on ICE*Kp* or IncFIB plasmid.

One of the key factors sustaining the successful global spread of carbapenemase encoding genes in *Enterobacteriaceae* is the transferability of plasmids^19^. The increase in carbapenem resistance is largely driven by self-conjugative plasmids^2^. Such plasmids carry multiple or single resistance determinants, such as *bla*_NDM-1_ and *bla*_OXA-48_^11^.

OXA-48 (61.8%; 21/34) and NDM (17.6%; 6/34) were the only two detected carbapenemases. Many other studies from Lebanon have reported that NDM-1 and OXA-48 are the only circulating carbapenemases in the country, but these completely focused on the detection of individual genes without looking into the core and pan-genome, mobilome, virulome, and resistome^20–23^. *bla*_OXA-48_ was detected in isolates belonging to different clones (15 different STs and 15 different K-types).

The highest number of detected Inc groups in one isolate was seven (KP1), presented as one big multi-replicon plasmid (IncFIIK, IncFIA, and IncR) and three relatively smaller plasmids each having a single replicon (IncA/C or IncX1, or IncL/M) as determined by PLACNETw. The IncP replicon was missed from the WGS data. Multi-replicon plasmids have been increasingly detected with IncF plasmids being the most common type showing this feature, and plasmids with three distinct replicons, IncFIIK, IncR, and IncFII, were previously described by Coelho *et al*.^19^. Interestingly, the multi-replicon plasmid detected in this study carried a *bla*_NDM-1_ gene. Since plasmids might occasionally be replaced or lost by an incoming incompatible plasmid, a multi-replicon status allows the acquisition of plasmids having incompatible replicons by driving its replication by a compatible one^12^. This entails a broader host range replication and is certainly alarming as it can further facilitate widespread dissemination^12^.

The detection of a functionally intact *bla*_CTX-M-14b_ gene in this study explains the resistance of KP11 to 2^nd^, 3^rd^, 4^th^, and non-extended spectrum cephalosporins, with the exception of ceftazidime, a phenotype that was not seen in the remaining ST45 isolates.

To date 20 NDM variants were reported, with *bla*_NDM-7_ being recently described^24^. This variant differed from NDM-1 by two amino acid substitutions at positions Asp-130-Asn and Met-154-Leu^25^. *bla*_NDM-7_ shows a higher hydrolysis efficiency against carbapenems compared to its ancestor *bla*_NDM-1_, which has a direct clinical and therapeutic implication, and often co-exists with other antibiotic resistance genes on the same IncX3 type conjugative plasmid^25^. IncX plasmids usually harbor either drug efflux system encoding genes or genes involved in resistance such as quinolone resistance, carbapenem resistance or extended-spectrum β-lactamases^8,26,27^. Unlike IncX1 and IncX4, the analysis of the complete genetic environment of *bla*_NDM-7_ on IncX3 in KP28 compared to a set of publicly available IncX3 plasmids revealed a typical backbone of IncX plasmids consisting of genes encoding replication (*repB*), partitioning (*parB*), maintenance (*topB*), and conjugal transfer (*pil* and *tax*) (Fig. 6)^8^. *bla*_NDM-7_ was carried by an IS*5-*$\triangle$IS*Aba125-bla*_NDM-7_*-ble*_MBL_*-trpF-dsbC*-IS*26* genetic element. A divergence in the genetic environment mobilizing the *bla*_NDM-7_ in KP28 IncX3 was highlighted through the absence of a second $\triangle$IS*Aba12*5 upstream of *bla*_NDM-7_, as previously evidenced in pKpN01-NDM7 plasmid^28^. Earlier studies infer that IncX3 pOM26-1 plasmid might have evolved from pKpN01-NDM7 plasmid by the loss of one $\triangle$IS*Aba125* element flanking IS*5*, which could also be the case for the identified plasmid in this study.

The genetic environment of the *bla*_NDM-1_ was also investigated, which in all isolates was carried on an IncF replicon in a chimeric structure with *ble*_MBL_ encoding bleomycin resistance^29^. The *bla*_NDM_*-ble*_MBL_ operon along with few neighboring genes was first formed in *Acinetobacter spp*. before being acquired by *Enterobacteriaceae*^30^. It is usually present on Tn*125* flanked by two $\triangle$IS*Aba125*^29^. The downstream $\triangle$IS*Aba125* was detected in all the isolates, whereas the upstream $\triangle$IS*Aba125* was absent due to Tn*3* insertion. This was also previously observed in *K. pneumoniae* and other *Enterobacteriaceae*^31^. Additionally, *bla*_NDM-1_ detected in KP18 was found on a multi-replicon plasmid possessing IncFIIK, IncFIB, and IncR, which were the only detected replicons. This suggested the presence of a single big plasmid carrying a considerable number of ARGs. IncR replicons detected in *K. pneumoniae* are commonly associated with a variety of ARGs such as *bla*_CTX-M-15_, *bla*_TEM-1_, *bla*_DHA-1_, *bla*_OXA-1_, *qnrS1, aac(6’)-Ib*, and *qnrB4*, all of which were found in KP18^2^. KP18 was typed as ST11, a SLV of ST258. ST258 is the predominant clone of KPC-producing *K. pneumoniae* and was responsible for its global dissemination^32^. ST11, sharing 80% of ST258 core genome, was also involved in the global spread of CTX-M-15 during the rise of ESBL-producing *K. pneumoniae,* and in recent years has acquired the *bla*_*NDM*-1_^33^. Accordingly, ST11 harboring *bla*_NDM-1_ is of a great concern since it has a superior epidemic potential and, once put under favorable conditions, can undergo quick clonal expansion and contribute to the successful global spread of *bla*_NDM-1_^7^.

Loss or alterations of major porins OmpK35 and OmpK36 coupled with ESBL production is also associated with carbapenem resistance^34^. The underlying mechanisms of resistance involve either alterations through point mutations that could lead to protein modifications or premature truncation, or through insertion sequences that could lead to porin gene interruption. In seven of the isolates showing carbapenem resistance, both porin encoding genes were altered through point mutations, with three causing the premature truncation of the OmpK36. Premature stop codons were only detected in the *ompK36* gene. Of note, all of these isolates were resistant to ertapenem, none showed resistance to meropenem and only one was resistant to imipenem. A similar finding was previously reported with carbapenem resistance involving porins and ESBLs being phenotypically observed against ertapenem only^35^. The presence of positive selection on these non-synonymous mutations was assessed using SNAP. Upon calculating the dN/dS ratio, it was shown that these mutations are positively selected for since the ratio was more than 1. Moreover, the overall number of non-synonymous mutations were shown to be much higher than that of synonymous mutations (Supplementary Fig. S1). This finding was expected since changes in porin proteins coupled with an ESBL or AmpC type of enzymes confer a survival advantage. Positive selection involving outer membrane porin proteins was also previously detected in *E. coli, Shigella flexneri, Neisseria gonorrhea* and *N. meningitidis*^36,37^.

The yersiniabactin siderophore, mobilized by the integrative and conjugative element ICE*Kp*, was one of the most common VFs found in almost half of the isolates in this study. The yersiniabactin locus was traced on diverse ICE*Kp* types with some being localized on a plasmid highly similar to *K. pneumoniae* INF167_p0001 plasmid (Supplementary Table S4)^17^. Unlike recent reports, none of the ICE*Kp* elements harbored a colibactin or salmochelin synthesis loci^17^. The observed diversity of the detected ICE*Kp* elements suggested its acquisition through horizontal gene transfer rather than stable vertical inheritance^17^. The presence of various iron-scavenging mechanisms can give rise to increased efficiency of iron uptake, which has been correlated with hypervirulent phenotypes^3^. Interestingly, KP25 carried a unique repertoire of VFs that included *kfu*, *all, glx*, *ybb*, and *ylbE*. The identification of *kfu* and *all* in KP25 with the capsular type K151 is a novel finding where it was previously only reported in strains having a K1 capsular type^38,39^*. all* is a relatively rare virulence gene found in organisms metabolizing allantoin to obtain carbon and nitrogen from the environment^4^.

One out of the three NDM-1 producing XDR isolates, KP16, remained susceptible to only one drug class. The eight detected XDR isolates (ST15, ST16, ST147 and ST995) harbored on the average two VFs, with the exception of KP16 that carried none. The convergence of ARGs and VFs is a particularly alarming phenomenon for the possible emergence of untreatable invasive *K. pneumoniae* infections.

Maximum-likelihood clustering based on core genome SNPs and recombination hotspots was better at showing the evolutionary relatedness of the isolates; the branch length indicates the number of substitutions that an isolate has undergone relative to a common ancestor.

Analyzing the recombination hotspots of the isolates revealed several sequences involved in transport and resistance. One hotspot was found to be allocated within the P-type HAD ATPase IC family sequence. The P-type HAD ATPase IC is a transmembrane pump for ion and lipid transportation linked to phospholipids modification; recently gaining attention as unique drug targets in fungi^40^. Similarly, 4-hydroxy-tetrahydrodipicolinate synthase (DHDPS) was another hotspot identified, which previously was identified as a potential drug target that can be used against *Mycobacterium tuberculosis*^41^. Recombination events in the *hipA/B* gene and the genes encoding the proteins of the Major Facilitator Superfamily detected in this study are also of prime importance due to them being involved in bacterial resistance to fluoroquinolones, β-lactams, and quaternary ammonium compounds^42,43^.

To our knowledge, this is the first comprehensive whole-genome-based characterization of carbapenem resistant *K. pneumoniae* in Lebanon, and the first detection and description of *K. quasipneumoniae* in the region. Few studies have addressed in details the genomic content of *K. quasipneumoniae* isolated from a clinical setting. These studies tackled hypervirulence in community-acquired *K. quasipneumoniae* isolates^44–46^. Even fewer studies have described MDR *K. quasipneumoniae*^47,48^. We were the first to identify and report the presence of carbapenem resistant *K. quasipneumoniae* in the Middle East. The multi-replicon nature of the detected plasmids, the observed diversity of STs and K-types, and the coexistence of specific VFs with a very high number of ARGs all indicate the possible emergence of a population of untreatable invasive *K. pneumoniae*. This is a problem that is too important to be ignored. WGS allowed us to explore in-depth the genetic variation within the *K. pneumoniae* population and the genetic relatedness of the highly resistant isolates. Within clinical settings of Lebanese hospitals, traditional methods of identification and typing, such as API20E, disk diffusion, or PFGE, are still commonly used. When compared to WGS, these methods are known to suffer from low resolution and discriminatory power. In this study, a genome-based workflow was coupled with these traditional methods to overcome their limitations and culminated in an accurate population snapshot of carbapenem resistant *K. pneumoniae* isolated from hospitalized patients in Lebanon. The obtained data can be exploited to limit the cross-transmission of such pathogens in hospital settings, prevent outbreaks, and global dissemination.

## Materials and Methods

### Ethical Approval

Ethical approval was not required as clinical isolates were collected and stored as part of routine clinical care. Clinical isolates and patient records/information were anonymous and de-identified prior to analysis.

### Bacterial isolates

A total of 32 *K. pneumoniae* and two *K. quasipneumoniae* isolates collected from 2012 to 2015 were recovered at the Clinical Microbiology Laboratory at AUBMC and were designated as KP1-KP32 and KQ1-KQ2, respectively. AUBMC is a 350-bed tertiary care teaching hospital. The isolates were collected from various body sites such as urine, deep throat, skin wounds, including one from a C-section, among others (Supplementary Table S2). The mean patients’ age was 58 ± 21 years old, with a range of 18 to 87 years with a 1:1 male to female ratio. Initial species identification was performed based on the API 20E system (bioMérieux, Marcy l’Etoile, France) and by the Matrix-Assisted Laser Desorption/Ionization Time of Flight (MALDI-TOF) system (Bruker Daltonik, GmbH, Bremen, Germany) following the manufacturer’s instructions.

### String test

The string test, a phenotypic screening test to assess the hypermucoviscous phenotype, was performed on fresh bacterial colonies, as previously described^3^. Colonies were considered positive if the viscous string was at least five mm in length as determined through stretching bacterial colonies on an agar plate using an inoculation loop.

### Antimicrobial testing

Antimicrobial susceptibility testing was performed by the disk diffusion technique on Mueller-Hinton agar and it included a panel of 26 antibiotic disks belonging to 15 classes (Supplementary Table S1). Additionally, carbapenems were tested by the E-test methodology (AB BIODISK, Solna, Sweden) to determine the minimal inhibitory concentrations (MICs) of ertapenem, imipenem, and meropenem. The obtained data was interpreted according to the CLSI guidelines (CLSI, 2017) and Galani *et al*.^14^.

### Bacterial DNA extraction

DNA extraction was performed from fresh bacterial colonies grown on TSA agar plates using the Nucleospin® Tissue kit (Macherey-Nagel, Germany) according to the manufacturer’s instructions. The extracted DNA was used for subsequent PCR assays and WGS.

### Detection of resistance genes by PCR assays

The most common ESBLs and carbapenemases encoding genes, *bla*_CTX-M_, *bla*_SHV,_ *bla*_IMP_*, bla*_VIM_*, bla*_KPC_*, bla*_NDM-1_, and *bla*_OXA-48-like_, were amplified as previously described (Supplementary Table S7).

### Plasmid replicon typing

Plasmid characterization was performed using the DIATHEVA PBRT kit (Diatheva, Fano, Italy) through a PCR-based replicon typing method consisting of eight multiplex PCR assays for the amplification of 25 replicons: A/C, B/O, FIA, FIB, FIB-M, FIC, FII, FIIK, FIIS, HI1, HI2, HIB-M, I1, I2, K, L/M, N, P, R, T, U, W, X1, X2, and Y found in the family *Enterobacteriaceae*. Positive controls were included for all reactions. All PCR reactions were performed according to the manufacturer’s instructions and visualized on a 2.5% agarose gel stained with ethidium bromide.

### PCR amplification and sequencing of *wzi* gene

To determine the capsular type, *wzi* gene typing was performed using *wzi*-TR and *wzi*-TF primers as previously described (Supplementary Table S7). K-types were assigned using the Institute Pasteur database (http://bigsdb.pasteur.fr/klebsiella). The isolates that were associated with multiple or no K-types by *wzi* sequencing were re-analyzed using the publicly available Kaptive online tool (https://github.com/katholt/Kaptive)^18^. Kaptive makes use of WGS data as input and assigns capsular types by analyzing the entire *cps* locus.

### Multilocus sequence typing (MLST)

MLST was performed as described on the Institute Pasteur MLST database targeting seven housekeeping genes (*rpoB, gapA, mdh, pgi, phoE, infB*, and *tonB*) using primers with universal sequencing tails. Genes were sequenced using the universal oF and oR primer pair. STs were assigned using the Institute Pasteur database. Novel STs were submitted to the curator and assigned as new designations (www.pasteur.fr/mlst).

### Pulsed-field gel electrophoresis (PFGE)

PFGE fingerprinting was performed using the XbaI restriction enzyme (ThermoScientific, Waltham, MA, USA), 1% SeaKem agarose gel, and the universal laboratory standard *Salmonella enterica subsp. enterica serovar Braenderup* (ATCC® BAA664™) according to the standard PulseNet protocol (http://www.pulsenetinternational.org). Electrophoresis was performed using the Bio-Rad laboratories CHEF DR-III system (Bio-Rad Laboratories, Bio-Rad Laboratories Inc., Hercules, CA, USA) with a run time of 12 h and switch time of 5–40 s (https://www.cdc.gov/pulsenet/). Gels were stained with ethidium bromide. For isolates showing identical pulsotypes or that were untypable by XbaI, PFGE was repeated using the secondary enzyme AvrI. PFGE profiles were analyzed with the BioNumerics software version 7.6.1 (Applied Maths, Sint-Martens-Latem, Belgium), with profiles assigned as different pulsotypes if three or more bands were different between the two of them. Pulsotypes were clustered based on the BioNumerics software analysis through dice correlation coefficients with an optimization of 1.5% and tolerance of 1.5%.

### Whole-genome sequencing

Genomic libraries were constructed using the Nextera XT DNA library preparation kit with dual indexing (Illumina). The libraries were sequenced on an Illumina MiSeq with 250 bp x 2 read length. Genome assembly was performed *de novo* using Spades Genome Assembler Version 3.6.0^49^. Quality control checks on the obtained raw sequence data was performed using FastQC version 0.11.5^50^.

### Genome assembly, annotation and analysis

The assembled genomes were annotated using the RAST online server (http://rast.nmpdr.org)^51^. The Comprehensive Antibiotic Resistance Database (CARD) and ResFinder 3.0 available on the Center for Genomic Epidemiology website (www.genomicepidemiology.org) were used to determine the presence of resistance genes^52,53^. The presence of putative VFs was screened using the VF scheme available on http://bigsdb.pasteur.fr. PlasmidFinder 1.3 was used to determine the presence of plasmids in the genomic sequences^54^. PLACNETw was used to identify and analyze plasmids starting from raw reads by creating a network of contig interactions upon assembly of reads with Velvet^55^. The sequences encoding plasmids were then extracted and aligned to references obtained from NCBI. PHASTER was used to identify the phage content of the isolates^56^. SNAP (http://www.hiv.lanl.gov) was used to calculate synonymous and non-synonymous substitution rates of the *ompK36* porin gene^57^.

### Comparative genome analysis

Plasmid sequences were extracted and aligned with corresponding reference strains using BioNumerics software version 7.6.1. A circular genome comparison was performed using BRIG^58^.

### Pan-genome and recombination analysis

Genomes were annotated using Prokka version 1.13. with a similarity cutoff e-value 10^−6^ and minimum contig size of 200 bp^59^. Annotated GFF3 files were piped into Roary version 3.12.0. choosing a minimum blastp identity of 95 and core gene prevalence in all (>99%) of the isolates^60^. A maximum-likelihood phylogenetic tree based on the core genome alignment was constructed using FastTree 2^61^. Recombination events in the core genes were assessed by Gubbins version 2.2.1 and a maximum-likelihood tree was generated using RAxML^62^. Eight reference *K. pneumoniae* genomes were included based on their ST, K-types, resistance profiles, and country of origin and included in the analysis to maximize diversity: KP_ST11_OXA-48 (accession # JNHB00000000.1), MGH 63 (accession # JJNB00000000), KGM-IMP216 (accession # LJOI01000001.1), HK787 (accession # NZ_CP006738.1), MGH 78578 (accession # CP000647.1), KpN01 (accession # CP012987.1), Kp_Goe_149473 (accession # CP018686.1), and LAU-KP1 (accession # AYQE00000000.1). The resulting phylogenetic tree, patient, and isolate metadata along with the pan-genome fingerprints of the isolates, core genome SNPs, and recombination hotspots were visualized on Phandango V 1.1.0^63^.

## Electronic supplementary material

Supplementary Materials

## Data Availability

This Whole Genome Shotgun project has been deposited in DDBJ/ENA/GenBank under the accession numbers listed in Supplementary Table S2.
