# Supplementary material for: Molecular Characterization of Carbapenem Resistant Klebsiella pneumoniae and Klebsiella quasipneumoniae Isolated from Lebanon
Source: Sci Rep. 2019 Jan 24;9:531. doi: 10.1038/s41598-018-36554-2 (PMC6345840; doi:10.1038/s41598-018-36554-2)
Supplement: Supplementary file 1 — Supplementary Materials [file 41598_2018_36554_MOESM1_ESM.pdf]

Molecular Characterization of Carbapenem Resistant *Klebsiella pneumoniae* and *Klebsiella quasipneumoniae* Isolated from Lebanon

Harout Arabaghian[1], Tamara Salloum[1], Sahar Alousi[1], Balig Panossian[1], George F. Araj[2] & Sima Tokajian[1,*]

[1]Department of Natural Sciences, School of Arts and Sciences, Lebanese American University, Byblos, 1401, Lebanon

[2]Department of Pathology and Laboratory Medicine, Faculty of Medicine, American University of Beirut Medical Center, Beirut, 1107, Lebanon

*Address for correspondence: Dr. Sima Tokajian, Department of Natural Sciences, School of Arts and Sciences, Lebanese American University, Byblos, 1401, Lebanon; Tel: +961-9-547254

Email: stokajian@lau.edu.lb

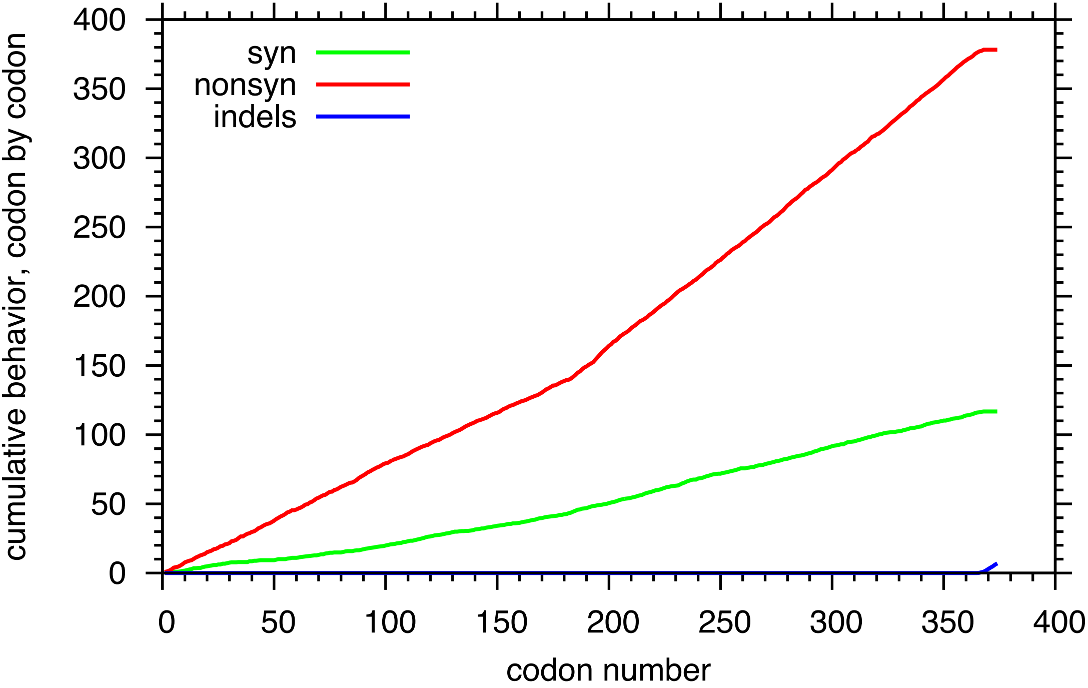

Supplementary Figure S1. OmpK36 cumulative dS/dN graph showing prevalence of cumulative synonymous and non-synonymous substitutions along the OmpK36 codon sequence. dS = 1.1621 dN = 1.4630 dN/dS = 1.2589

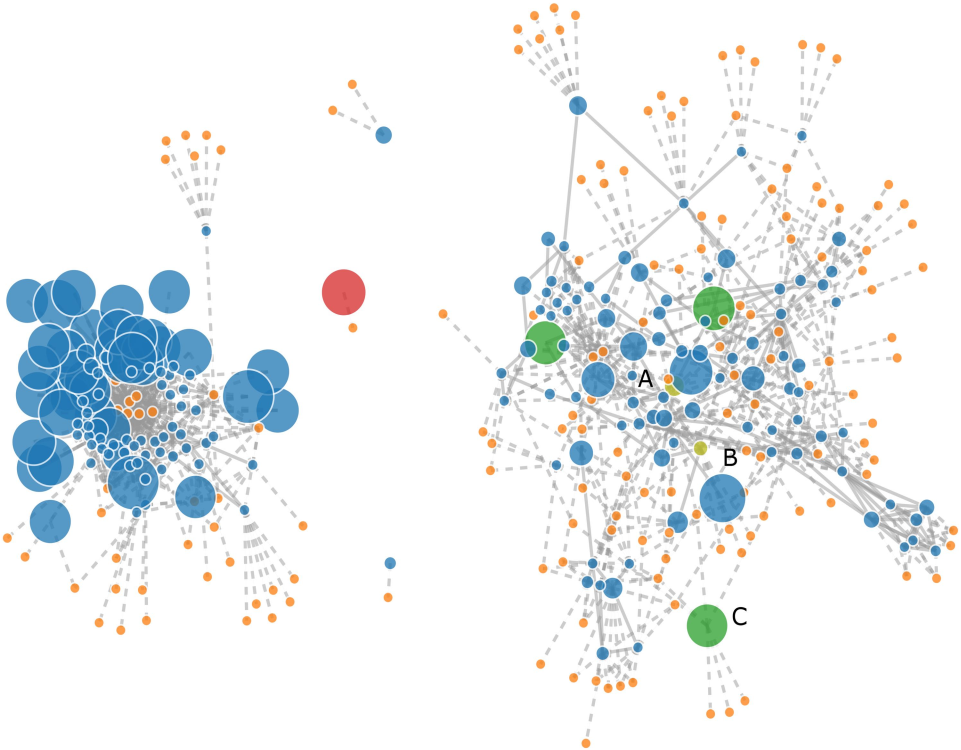

Supplementary Figure S2. Schematic representation of the KP1 genome using PLACNETw. KP1 contains a single multi-replicon plasmid (containing three replicons A, B, and C) and three smaller plasmids. A: IncFIA, B: IncR, C: IncFIIK, green circles: relaxase and replication initiator; red circle: relaxase; yellow circles: replication intitiator; blue circles: contigs.

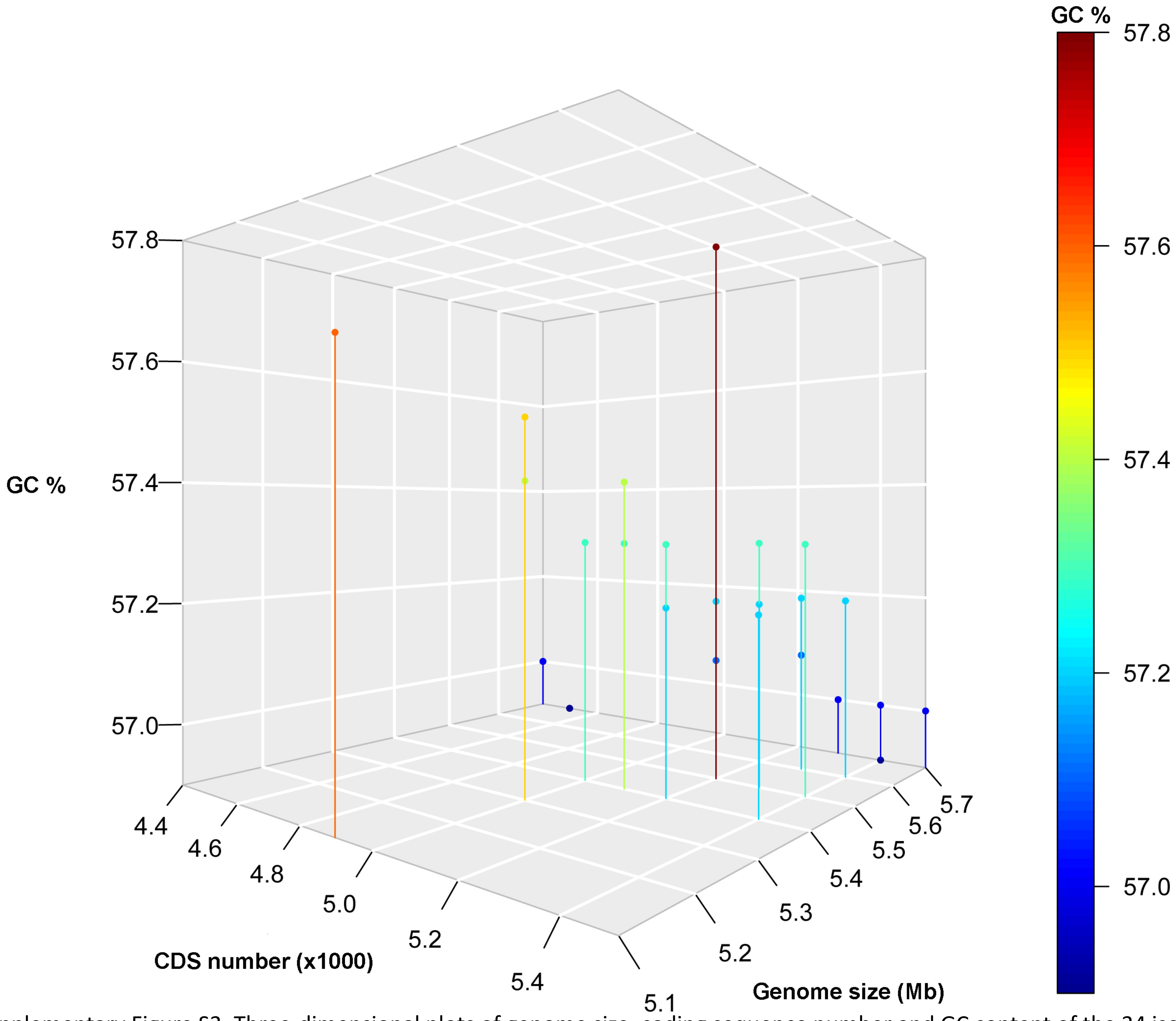

Supplementary Figure S3. Three-dimensional plots of genome size, coding sequence number and GC content of the 34 isolates; CDSs: number of coding sequences.

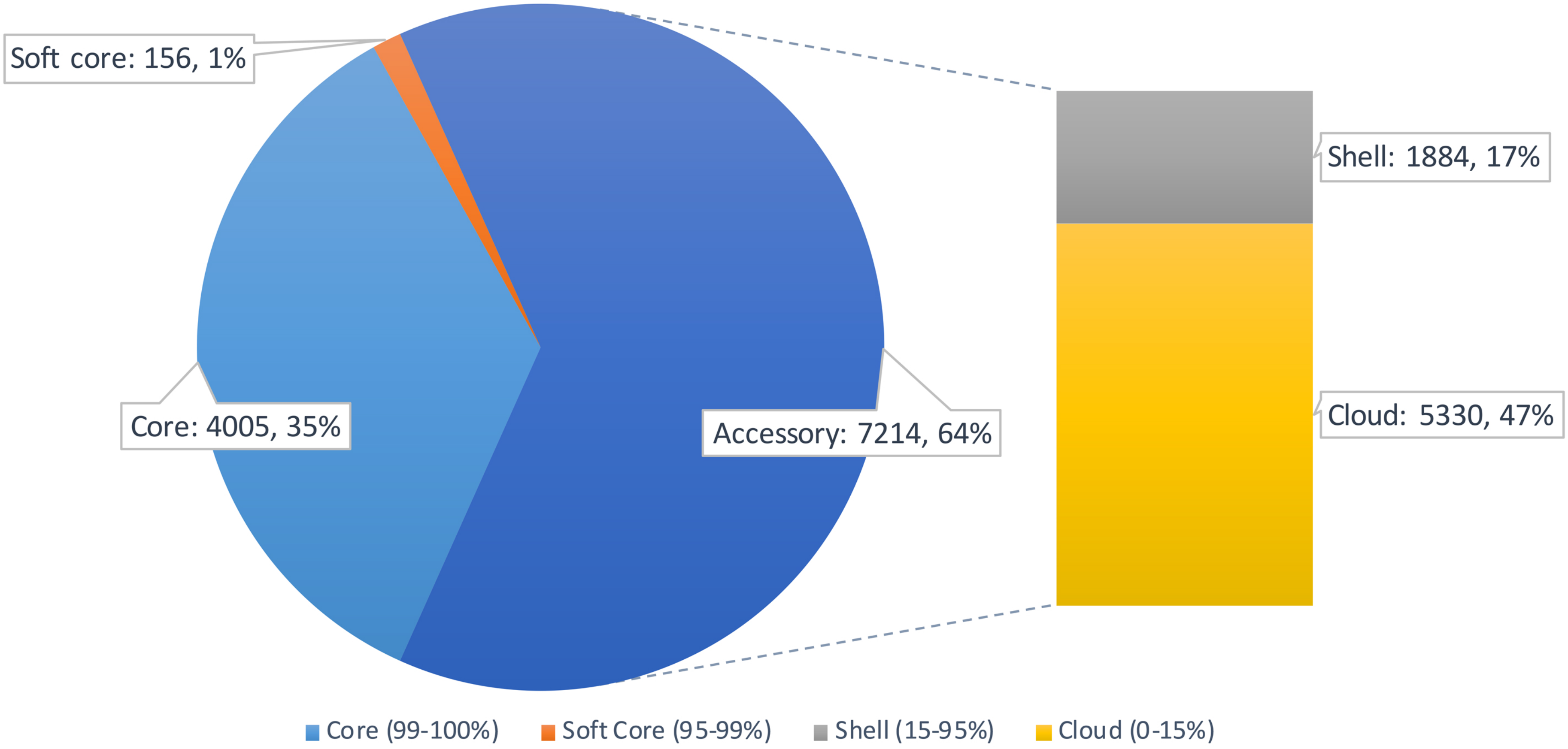

Supplementary Figure S4. Gene distribution in *K. pneumoniae* pan-genome.

Supplementary Table S1a. Antimicrobial susceptibility testing results for the tested antibiotics against all of the isolates.

Isolate	Aminoglycosides		Penicillin	Penicillin + b-lactamase inhibitor		Monobactam	Non-extended Cephalosporin	Extended-spectrum Cephalosporin					Cephamycin
								2nd generation Cephalosporins	4th generation Cephalosporins	3rd generation Cephalosporins			
	Amikacin	Gentamicin	Ampicillin	Augmentin	Tazobactam	Aztreonam	Cefuroxime	Cefamandole	Cefepime	Ceftazidime	Cefotaxime	Cefixime	Cefoxitin
KP1	R	R	R	R	R	R	R	R	R	R	R	R	R
KP2	S	S	R	I	I	R	R	R	R	R	R	R	R
KP3	S	S	R	R	R	R	R	R	S	R	R	R	S
KP4	S	S	R	R	R	S	I	I	S	S	S	S	S
KP5	S	S	R	R	R	R	R	R	S	R	R	S	S
KP6	S	S	R	R	R	R	R	R	R	R	R	R	S
KP7	S	R	R	I	I	R	R	R	R	R	R	R	R
KP8	S	R	R	I	R	R	R	R	R	R	R	R	I
KP9	S	S	R	R	R	S	I	I	S	S	S	S	S
KP10	S	R	R	R	R	R	R	R	S	R	R	R	S
KP11	S	S	R	R	R	I	R	R	I	S	R	R	R
KP12	S	R	R	R	R	R	R	R	R	R	R	R	S
KP13	S	R	R	R	R	R	R	R	R	R	R	R	S
KP14	S	S	R	R	R	S	I	I	S	S	I	S	S
KP15	S	S	R	R	R	S	R	R	S	S	R	S	R
KP16	R	R	R	R	R	R	R	R	R	R	R	R	R
KP17	S	S	R	R	R	R	R	R	R	R	R	R	R
KP18	S	S	R	R	S	R	R	R	R	R	R	R	R
KP19	S	S	R	R	R	S	I	I	S	S	S	S	S
KP20	R	R	R	R	R	R	R	R	S	R	R	R	R
KP21	S	R	R	I	S	R	R	R	R	R	R	S	S
KP22	R	R	R	R	R	S	R	R	R	R	R	R	R
KP23	S	S	R	R	R	S	S	S	S	S	S	S	S
KP24	S	S	R	R	R	S	I	I	S	S	I	S	S
KP25	S	R	R	R	R	S	S	S	S	S	S	S	S
KP26	S	S	R	R	R	S	S	S	S	S	I	S	S
KP27	S	S	R	R	S	R	R	R	R	R	R	R	R
KP28	S	S	R	R	R	R	R	R	R	R	R	R	R
KP29	S	S	R	R	R	S	S	S	S	S	S	S	S
KP30	R	R	R	R	R	R	R	R	R	R	R	R	R
KP31	S	S	R	R	R	S	S	S	S	S	S	S	S
KP32	S	R	R	I	I	R	R	R	R	R	R	R	R
KQ1	S	S	R	R	R	S	S	S	S	S	S	S	S
KQ2	S	S	R	R	R	S	S	S	S	S	S	S	S

Supplementary Table S1b. Antimicrobial susceptibility testing results for the tested antibiotics against all of the isolates.

Isolate	Fluoroquinolones				Nitrofurans	Tetracycline	Folate pathway inhibitors	Carbapenems			Phosphonic acids	Polymyxins	Glycyclines
	Ciprofloxacin	Levofloxacin	Norfloxacin	Ofloxacin	Nitrofurantoin	Tetracycline	Trimethoprim	Ertapenem	Imipenem	Meropenem	Fosfomycin	Colistin	Tigecycline
KP1	R	R	R	R	R	R	R	R	R	R	S	S	I
KP2	R	R	R	R	R	R	R	R	S	S	I	S	I
KP3	R	R	R	R	R	R	R	R	R	I	S	S	S
KP4	S	S	S	S	R	S	S	R	R	I	S	S	S
KP5	I	I	S	R	I	S	S	R	R	I	S	I	S
KP6	S	S	R	S	R	S	S	I	I	I	S	I	S
KP7	R	R	R	R	R	R	R	R	S	I	R	S	S
KP8	R	R	R	R	I	R	R	R	S	S	S	S	S
KP9	S	S	S	S	S	S	S	R	I	S	R	S	S
KP10	I	I	I	I	S	R	R	I	I	S	S	S	S
KP11	S	S	S	S	S	R	R	R	I	S	S	S	I
KP12	R	R	R	R	S	R	R	R	R	S	S	S	S
KP13	R	R	R	R	S	S	R	R	R	I	S	S	S
KP14	S	S	S	S	R	S	S	R	R	R	S	S	S
KP15	S	S	S	S	R	S	S	R	R	R	R	I	I
KP16	R	R	R	R	R	R	R	R	R	R	S	I	S
KP17	S	S	S	S	R	S	R	R	R	R	S	I	S
KP18	R	R	R	R	R	R	R	R	R	R	S	I	S
KP19	S	S	S	S	I	S	S	R	R	R	S	S	S
KP20	R	R	S	S	R	R	R	R	R	S	S	S	S
KP21	I	I	S	S	R	R	R	I	S	S	S	S	S
KP22	S	S	S	S	R	S	S	R	R	R	S	S	S
KP23	S	S	S	S	S	S	S	R	R	I	S	S	S
KP24	S	S	S	S	S	S	S	R	R	I	S	I	S
KP25	S	S	S	S	S	R	R	I	R	S	S	S	S
KP26	S	S	S	S	R	S	S	R	R	I	S	S	S
KP27	R	R	R	R	R	R	R	R	R	I	S	I	R
KP28	R	R	R	R	R	R	R	R	R	R	S	I	I
KP29	I	S	S	S	S	S	R	R	R	R	S	I	S
KP30	R	R	R	R	R	R	R	R	R	S	S	I	I
KP31	S	S	S	S	R	S	S	R	R	I	S	S	S
KP32	R	R	R	R	R	R	R	R	S	I	S	S	R
KQ1	S	S	S	S	S	R	S	I	I	I	S	S	S
KQ2	S	S	S	S	S	R	S	R	R	I	S	S	S

Supplementary Table S2. Isolate information including source of specimen, sex and age of patients, year of isolation, MDR or XDR status and NCBI accession numbers.

Isolate	Source	Sex	Age	Year	MDR or XDR status	Accession #
KP1	DTA	M	75	2012	XDR	NIBY00000000
KP2	C Section Wound	F	38	2012	XDR	NIBZ00000000
KP3	Urine	F	18	2013	MDR	NICA00000000
KP4	Urine	F	87	2013	MDR	NICB00000000
KP5	DTA	M	67	2013	MDR	NICC00000000
KP6	Cyst	M	20	2013	MDR	NICD00000000
KP7	Drain Site	F	83	2013	XDR	NICE00000000
KP8	Drain Site	F	83	2013	MDR	NICF00000000
KP9	Urine	F	63	2013	MDR	NICG00000000
KP10	Urine	F	71	2013	MDR	NICH00000000
KP11	Lung Swab	M	69	2013	MDR	NICI00000000
KP12	DTA	F	79	2013	MDR	NIFL00000000
KP13	DTA	F	79	2013	MDR	NIFM00000000
KP14	Urine	M	72	2013	MDR	NICJ00000000
KP15	Urine	M	73	2013	MDR	NICK00000000
KP16	Unknown	F	82	2013	XDR	NICL00000000
KP17	Urine	F	33	2013	MDR	NICM00000000
KP18	DTA	M	59	2013	MDR	NICN00000000
KP19	Urine	M	68	2013	MDR	NICO00000000
KP20	Leg Tissue	M	22	2013	MDR	NICP00000000
KP21	Incision Site Swab	M	32	2014	MDR	NICQ00000000
KP22	Skin	F	50	2014	MDR	NICR00000000
KP23	Skin	M	64	2014	MDR	NICU00000000
KP24	Peripheral	M	54	2015	MDR	NICV00000000
KP25	Urine	F	33	2015	MDR	NICW00000000
KP26	Wound	F	74	2015	MDR	NICX00000000
KP27	Urine	M	30	2015	XDR	NICY00000000
KP28	Urine	M	74	2015	XDR	NICZ00000000
KP29	Skin	M	59	2015	MDR	NWBX00000000
KP30	Urine	F	57	2013	XDR	NWBY00000000
KP31	Skin	M	62	2015	MDR	NWBZ00000000
KP32	Urine	M	30	2015	XDR	NWCA00000000
KQ1	Isolate	F	36	2014	MDR	NICS00000000
KQ2	Pus	F	79	2014	MDR	NICT00000000

Supplementary Table S3. Virulence determinants present in the isolates and results of the string test; LPS: lipopolysaccharide.

Sample	Siderophore														Adhesins and Biofilm									Others							Micorcin									LPS	Hypermucoviscous		Regulator		String Test
	Yersiniabactin											Iron uptake			Fimbriae								Pili (Twitching motility)																						
	ybtA	*ybtE*	*ybtP*	*ybtQ*	*ybtS*	*ybtT*	*ybtU*	*ybtX*	*fyuA*	*irp1*	*irp2*	*kfuA*	*kfuB*	*kfuC*	*mrkA*	*mrkB*	*mrkC*	*mrkD*	*mrkF*	*mrkH*	*mrkI*	*mrkJ*	*pili*	*allC*	*allD*	*allS*	*glxK*	*glxR*	*ybbW*	*ylbE*	*mceA*	*mceB*	*mceC*	*mceD*	*mceE*	*mceG*	*mceH*	*mceI*	*mceJ*	*waa*	*rmpA*	*rmpA2*	*kvgA*	*kvgS*	
**KP1**																																													
**KP2**																																													
**KP3**																																													
**KP4**																																													
**KP5**																																													
**KP6**																																													
**KP7**																																													
**KP8**																																													
**KP9**																																													
**KP10**																																													
**KP11**																																													
**KP12**																																													
**KP13**																																													
**KP14**																																													
**KP15**																																													
**KP16**																																													
**KP17**																																													
**KP18**																																													
**KP19**																																													
**KP20**																																													
**KP21**																																													
**KP22**																																													
**KP23**																																													
**KP24**																																													
**KP25**																																													
**KP26**																																													
**KP27**																																													
**KP28**																																													
**KP29**																																													
**KP30**																																													
**KP31**																																													
**KP32**																																													
**KQ1**																																													
**KQ2**																																													

Supplementary Table S4. Description of ICE*Kp* variants detected among the isolates.

**ICE*Kp***	**Isolate**	**YbST lineages from Lam et al (2018)**	**Length (kbp)**	**Unique genes in variable regions from Lam et al (2018)**	**Accession number**
ICE*Kp3*	KP17, KP20	8, 9	65	Restriction endonuclease, DUF4917 domain containing protein, ATP/GTP phosphatase, Reverse transcriptase, DDE endonuclease, 5 Hypothetical proteins	KY454628
ICE*Kp4*	KP1-3, KP11-13, KP25, KP28-31, KP34	10	58	Transposase, ABC transporter, Type I restriction endonuclease, DNA methyltransferase, Hypothetical protein	KY454629
ICE*Kp5*	KP10	6, 14	66	DEAD/DEAH box helicase, Thiamine biosynthesis protein ThiF, 2 Patatin-like phospholipases, 6 Hypothetical proteins	KY454630
ICE*Kp12*	KP19	9, 10, 14, 16	97	Exonuclease SbcC, DNA helicase UvrD, ATP-dependent endonuclease, 5 Hypothetical proteins	KY454636

Supplementary Table S5. Genome statistics. The number of subsystems was inferred by RAST annotation tool; CDSs: coding sequences; STDEV: standard deviation.

#	Sample	Coverage	Size	GC %	N50	L50	Contigs	Subsystems	CDSs	RNAs
1	KP1	30x	5,701,879	57	383386	6	90	595	5426	110
2	KP2	30x	5,538,655	57.3	365966	5	92	598	5299	110
3	KP3	30x	5,483,149	57.2	298205	7	83	592	5184	112
4	KP4	30x	5,345,213	57.4	393139	6	80	590	5057	125
5	KP5	30x	5,624,416	57.1	350416	5	113	595	5332	121
6	KP6	30x	5,617,544	57.1	356543	5	88	595	5334	120
7	KP7	30x	5,418,820	57.2	377281	5	73	596	5213	115
8	KP8	30x	5,400,670	57.3	379465	4	69	596	5194	115
9	KP9	30x	5,500,715	57.2	305666	6	68	598	5216	106
10	KP10	30x	5,704,986	57	341307	5	114	599	5478	111
11	KP11	30x	5,552,392	57.2	319350	6	98	592	5336	112
12	KP12	30x	5,732,024	56.9	294939	8	100	598	4550	106
13	KP13	30x	5,674,785	57	294939	8	95	596	5371	106
14	KP14	30x	5,178,076	57.6	420528	4	84	586	4920	119
15	KP15	30x	5,409,266	57.2	350623	6	78	589	5156	112
16	KP16	30x	5,545,493	57	419,656	6	80	594	5,313	114
17	KP17	30x	5,428,356	57.4	351,158	6	94	594	5,116	118
18	KP18	30x	5,547,879	57.3	328,662	6	118	598	5,361	105
19	KP19	30x	5,442,987	57.2	328,518	7	97	587	5,172	120
20	KP20	30x	5,359,526	57.3	438631	3	48	588	5,072	102
21	KP21	30x	5,508,229	57.1	315,823	5	116	588	5,184	110
22	KP22	30x	5,726,784	57	355,645	6	135	598	5,468	100
23	KP23	30x	5,396,502	57.4	759,858	3	83	590	5,121	119
24	KP24	30x	5,389,400	57.3	364,756	6	82	589	5,094	104
25	KP25	30x	5,412,803	57.4	482,897	4	91	592	5,147	121
26	KP26	30x	5,568,664	57.2	369,998	5	88	598	5,274	129
27	KP27	30x	5,655,394	57	294,938	8	94	596	5,336	114
28	KP28	30x	5,600,423	57.2	425,815	3	102	598	5,382	113
29	KP29	30x	5,612,182	57.2	289,762	7	100	599	5,340	107
30	KP30	30x	5,279,237	57.5	354,225	5	84	587	4991	115
31	KP31	30x	5,680,583	56.9	255933	9	115	596	5385	114
32	KP32	30x	5,525,132	57.2	259,569	6	108	595	5,280	131
33	KQ1	30x	5,495,455	57.8	473,825	4	93	595	5,207	110
34	KQ2	30x	5,490,427	57.8	473,824	4	83	595	5,190	111
		Average	5,516,119	57	369,860	6	92	594	5,221	113
		STDEV	134003.036	0.216	90349.921	1.48101473	16.9760238	3.95248066	178.15	7.2203

Supplementary Table S6. Phage content in the 34 isolates. In total, 43 different phages were identified. The number of detected phages ranged from 1 in KP14 and KP29 to 11 phages in KP18. Salmonella phage RE-2010 (accession no. NC_019488) was the most commonly identified in 38.2% (n=13) of the isolates.

	#	Region	Completeness	# of Proteins	Position	Phage	GC %	Accession #
KP1	1	55.2Kb	intact	49	914751-970036	Enterobacteria phage mEp237	51.34%	NC_019704
	2	47Kb	intact	51	3380502-3427508	Salmonella phage 118970_sal3	51.12%	NC_031940
	3	40.2Kb	intact	49	3645014-3685272	Bacteriophage 186	52.39%	NC_001317
	4	18.4Kb	incomplete	23	4810228-4828690	Enterobacteria phage P1	54.96%	NC_005856
	5	79.2Kb	intact	101	5331098-5410390	Klebsiella phage phiKO2	50.70%	NC_005857
KP2	1	48.4Kb	intact	59	712379-760819	Cronobacter phage ENT47670	51.76%	NC_019927
	2	46.4Kb	intact	73	1603259-1649724	Salmonella phage SEN34	53.81%	NC_028699
	3	38.3Kb	intact	38	4298937-4337258	Salmonella phage SEN5	50.06%	NC_028701
	4	82.1Kb	intact	84	4457324-4539481	Enterobacteria phage mEp237	52.78%	NC_019704
	5	38.6Kb	incomplete	35	5200991-5239607	Salmonella phage Fels-1	51.76%	NC_010391
	6	35.6Kb	intact	44	5455142-5490762	Enterobacteria phage HK140	54.27%	NC_019710
	7	23.8Kb	incomplete	16	5502376-5526273	Pseudomonas phage YMC11/07/P54_PAE_BP	55.67%	NC_030909
KP3	1	71.5Kb	intact	62	1742074-1813596	Edwardsiella phage GF-2	55.56%	NC_026611
	2	27.4Kb	incomplete	17	3014842-3042269	Salmonella phage 118970_sal3	54.32%	NC_031940
	3	37.8Kb	intact	40	3366089-3403943	Salmonella phage SEN5	50.42%	NC_028701
	4	48.4Kb	intact	49	3530379-3578820	Acinetobacter phage vB_AbaS_TRS1	50.61%	NC_031098
	5	22.6Kb	intact	28	4817281-4839974	Enterobacteria phage HK544	51.38%	NC_019767
KP4	1	27.1Kb	incomplete	29	102698-129891	Escherichia phage HK639	52.28%	NC_016158
	2	22.7Kb	intact	28	1794236-1817032	Enterobacteria phage P88	57.06%	NC_026014
	3	24.1Kb	intact	25	4790176-4814373	Enterobacteria phage P88	53.64%	NC_026014
KP5	1	51.1Kb	intact	44	869628-920735	Salmonella phage 118970_sal3	52.10%	NC_031940
	2	32Kb	intact	38	3321812-3353900	Bacteriophage 186	53.40%	NC_001317
	3	22.4Kb	questionable	20	3708615-3731029	Acinetobacter phage vB_AbaS_TRS1	52.27%	NC_031098
	4	36.1Kb	intact	41	4458293-4494443	Salmonella phage SEN5	54.72%	NC_028701
KP6	1	47.8Kb	intact	37	965640-1013538	Salmonella phage 118970_sal3	51.97%	NC_031940
	2	32Kb	intact	39	3464346-3496434	Bacteriophage 186	53.40%	NC_001317
	3	33.4Kb	incomplete	27	4095088-4128579	Enterobacteria phage mEp237	52.05%	NC_019704
	4	21Kb	questionable	20	4129167-4150245	Acinetobacter phage vB_AbaS_TRS1	52.80%	NC_031098
	5	36.1Kb	intact	40	4720240-4756390	Salmonella phage SEN5	54.72%	NC_028701
	6	22.1Kb	incomplete	24	5284859-5307029	Cronobacter phage ENT39118	55.66%	NC_019934
KP7	1	53.1Kb	intact	53	74086-127279	Enterobacteria phage mEp237	52.06%	NC_019704
	2	77.2Kb	intact	76	1075415-1152644	Salmonella phage SEN34	52.38%	NC_028699
	3	37.6Kb	intact	45	2287591-2325203	Salmonella phage 118970_sal3	52.11%	NC_031940
	4	40Kb	intact	45	4306499-4346539	Salmonella phage 118970_sal3	51.36%	NC_031940
	5	38.3Kb	intact	36	4746607-4784928	Salmonella phage SEN4	50.06%	NC_029015
	6	50.1Kb	intact	53	5125887-5175991	Salmonella phage SJ46	51.45%	NC_031129
	7	36.4Kb	incomplete	58	5367227-5403722	Salmonella phage SEN34	52.42%	NC_028699
KP8	1	38.3Kb	intact	40	42298-80619	Salmonella phage SEN4	50.06%	NC_029015
	2	53.1Kb	intact	55	205236-258429	Enterobacteria phage mEp237	52.06%	NC_019704
	3	77.2Kb	intact	75	1206565-1283794	Salmonella phage SEN34	52.38%	NC_028699
	4	40.4Kb	intact	41	2762740-2803152	Salmonella phage 118970_sal3	52.17%	NC_031940
	5	44.5Kb	intact	50	4436414-4480967	Salmonella phage 118970_sal3	52.04%	NC_031940
	6	16.7Kb	questionable	21	5119886-5136657	Staphylococcus phage SPbeta-like	51.80%	NC_029119
	7	42.3Kb	intact	48	5136658-5178994	Enterobacteria phage P1	50.80%	NC_005856
	8	11.5Kb	incomplete	22	5359394-5370913	Salmonella phage SEN34	52.57%	NC_028699
KP9	1	9.1Kb	incomplete	10	762750-771903	Sphingomonas phage PAU	52.55%	NC_019521
	2	44.6Kb	intact	45	1873891-1918497	Enterobacteria phage Fels-2	52.23%	NC_010463
	3	40.6Kb	intact	43	2443769-2484424	Salmonella phage RE-2010	53.48%	NC_019488
	4	44.7Kb	intact	45	3764659-3809374	Enterobacteria phage HK140	50.16%	NC_019710
KP10	1	22.7Kb	intact	28	2480564-2503279	Enterobacteria phage P88	57.39%	NC_026014
	2	47.8Kb	intact	56	2818001-2865810	Salmonella phage SPN1S	54.45%	NC_016761
	3	13.1Kb	incomplete	14	3832622-3845783	Enterobacteria phage P4	51.38%	NC_001609
	4	13.7Kb	questionable	15	5040370-5054090	Enterobacteria phage P4	48.29%	NC_001609
	5	31.1Kb	intact	30	5095297-5126468	Salmonella phage SJ46	51.77%	NC_031129
	6	22.8Kb	incomplete	26	5343788-5366648	Salmonella phage 118970_sal3	53.19%	NC_031940
	7	29.4Kb	questionable	40	5420801-5450260	Acinetobacter phage vB_AbaS_TRS1	52.22%	NC_031098
	8	64.3Kb	intact	60	5487736-5552117	Enterobacteria phage mEpX1	51.17%	NC_019709
KP11	1	21.7Kb	incomplete	24	2187029-2208797	Cronobacter phage ENT39118	53.60%	NC_019934
	2	30.6Kb	incomplete	37	4699164-4729819	Escherichia phage HK639	54.68%	NC_016158
	3	37.5Kb	intact	41	4861980-4899526	Salmonella phage SEN4	50.56%	NC_029015
	4	63.2Kb	intact	68	5190949-5254177	Klebsiella phage phiKO2	53.15%	NC_005857
	5	25.2Kb	incomplete	43	5362240-5387505	Escherichia phage HK639	53.93%	NC_016158
	6	28.4Kb	questionable	33	5401383-5429872	Salmonella phage SEN34	53.28%	NC_028699
KP12	1	35.8Kb	intact	43	3944827-3980649	Salmonella phage RE-2010	51.46%	NC_019488
	2	127.8Kb	intact	108	4625026-4752860	Salmonella phage SSU5	48.68%	NC_018843
	3	6.4Kb	incomplete	6	5493198-5499655	Salmonella phage SJ46	58.73%	NC_031129
	4	8.3Kb	incomplete	15	5722930-5731232	Enterobacteria phage P1	53.62%	NC_005856
KP13	1	35.8Kb	intact	44	3916783-3952605	Salmonella phage RE-2010	51.46%	NC_019488
	2	118.1Kb	intact	109	4617822-4736016	Salmonella phage SSU5	49.79%	NC_018843
KP14	1	40.9Kb	intact	43	705153-746065	Salmonella phage 64795_sal3	51.95%	NC_031918
	1	46.6Kb	intact	61	2194448-2241101	Cronobacter phage ENT47670	52.93%	NC_019927
	2	35.2Kb	incomplete	24	4429646-4464903	Enterobacteria phage mEp237	53.66%	NC_019704

KP15	3	121.9Kb	intact	125	4774724-4896641	Salmonella phage SSU5	49.10%	NC_018843
	4	20.8Kb	incomplete	31	5183005-5203827	Cronobacter phage ENT39118	53.67%	NC_019934
	5	44.2Kb	intact	53	5318971-5363225	Acinetobacter phage vB_AbaS_TRS1	51.51%	NC_031098
	1	55.4Kb	intact	51	481459-536858	Enterobacteria phage mEp237	51.42%	NC_019704
	2	52.7Kb	intact	62	3253236-3305941	Cronobacter phage ENT47670	53.11%	NC_019927
KP16	3	40.2Kb	intact	49	3663702-3703960	Bacteriophage 186	52.40%	NC_001317
	4	16.8Kb	incomplete	22	3962833-3979651	Pectobacterium phage ZF40	48.86%	NC_019522
	5	119.4Kb	intact	117	4758652-4878141	Salmonella phage SSU5	49.34%	NC_018843
KP17	1	35.9Kb	intact	44	1038682-1074619	Salmonella phage RE-2010	51.36%	NC_019488
	2	40.1Kb	intact	42	3942507-3982673	Salmonella phage Fels-1	50.93%	NC_010391
	1	21.2Kb	incomplete	18	1415424-1436698	Salmonella phage RE-2010	49.70%	NC_019488
	2	21.8Kb	incomplete	10	1750215-1772106	Enterobacteria phage P4	55.08%	NC_001609
	3	31.4Kb	intact	28	3417132-3448580	Bacteriophage 186	53.85%	NC_001317
	4	33Kb	questionable	25	3631870-3664923	Salmonella phage ST64B	52.24%	NC_004313
	5	30.8Kb	incomplete	28	4507332-4538222	Salmonella phage SEN34	54.02%	NC_028699
KP18	6	30.6Kb	intact	23	5048230-5078877	Stx2-converting phage 1717	56.65%	NC_011357
	7	35.4Kb	questionable	27	5064277-5099726	Enterobacteria phage P4	54.16%	NC_001609
	8	20.4Kb	incomplete	9	5153242-5173669	Staphylococcus phage SPbeta-like	56.00%	NC_029119
	9	20.2Kb	questionable	23	5237237-5257492	Enterobacteria phage Fels-2	51.34%	NC_010463
	10	19.2Kb	intact	24	5379811-5399086	Salmonella phage RE-2010	53.35%	NC_019488
	11	42.3Kb	intact	58	5439737-5482095	Salmonella phage RE-2010	54.43%	NC_019488
	1	36.2Kb	intact	38	249214-285490	Enterobacteria phage mEp235	50.44%	NC_019708
KP19	2	28.5Kb	intact	30	4376629-4405226	Acinetobacter phage vB_AbaS_TRS1	51.98%	NC_031098
	3	31.1Kb	incomplete	23	4602541-4633696	Enterobacteria phage mEp237	52.90%	NC_019704
	1	35Kb	questionable	35	1081988-1116990	Acinetobacter phage vB_AbaS_TRS1	51.39%	NC_031098
	2	24Kb	intact	26	1509308-1533316	Enterobacteria phage P88	53.76%	NC_026014
KP20	3	10.2Kb	incomplete	11	1848616-1858910	Salmonella phage RE-2010	48.00%	NC_019488
	4	50.7Kb	intact	56	2299299-2350077	Cronobacter phage ENT47670	52.78%	NC_019927
	5	40.6Kb	intact	49	4694637-4735287	Salmonella phage RE-2010	53.05%	NC_019488
	1	49Kb	intact	63	305908-354919	Salmonella phage vB_SosS_Oslo	52.46%	NC_018279
KP21	2	52Kb	intact	34	3394810-3446894	Enterobacteria phage mEp235	52.27%	NC_019708
	3	59.1Kb	intact	77	5047086-5106208	Salmonella phage SPN3UB	52.26%	NC_019545
	4	11.2Kb	incomplete	16	5430197-5441396	Enterobacteria phage phi92	51.38%	NC_023693
	1	34.1Kb	incomplete	22	490767-524912	Pectobacterium phage ZF40	52.47%	NC_019522
	2	34.5Kb	intact	25	1292141-1326660	Klebsiella phage phiKO2	53.74%	NC_005857
KP22	3	48.6Kb	intact	56	1918949-1967588	Edwardsiella phage GF-2	52.13%	NC_026611
	4	37.4Kb	intact	45	2275380-2312796	Enterobacteria phage Fels-2	50.62%	NC_010463
	5	26.5Kb	intact	31	4920636-4947220	Enterobacteria phage mEp043 c-1	50.37%	NC_019706
	6	14.8Kb	intact	13	5275095-5289926	Stx2-converting phage 1717	52.10%	NC_011357
KP23	1	34.7Kb	intact	41	101013-135760	Enterobacteria phage P88	53.94%	NC_026014
	2	111Kb	intact	105	4778689-4889726	Salmonella phage SSU5	48.92%	NC_018843
	1	23.9Kb	intact	25	56462-80410	Enterobacteria phage P88	53.86%	NC_026014
KP24	2	23.2Kb	questionable	20	229001-252292	Acinetobacter phage vB_AbaS_TRS1	51.85%	NC_031098
	3	23.8Kb	incomplete	14	2580143-2603995	Enterobacteria phage Sf6	49.05%	NC_005344
	1	53.3Kb	intact	52	1211269-1264591	Enterobacteria phage mEp237	51.12%	NC_019704
	2	7.9Kb	incomplete	8	1329989-1337897	Klebsiella phage 0507-KN2-1	47.38%	NC_022343
KP25	3	39.8Kb	intact	48	3993961-4033835	Salmonella phage RE-2010	52.83%	NC_019488
	4	35.9Kb	incomplete	24	5231415-5267344	Enterobacteria phage P1	53.93%	NC_005856
	5	8.3Kb	incomplete	8	5383778-5392150	Enterobacteria phage phi92	55.15%	NC_023693
	1	7Kb	incomplete	10	430842-437895	Pseudomonas phage PAJU2	54.08%	NC_011373
	2	52.6Kb	intact	46	4330389-4383074	Enterobacterial phage mEp213	52.07%	NC_019720
KP26	3	42.9Kb	intact	41	4725111-4768013	Enterobacteria phage mEp043 c-1	52.76%	NC_019706
	4	42.4Kb	intact	34	4996998-5039452	Klebsiella phage phiKO2	52.15%	NC_005857
	5	14.2Kb	incomplete	26	5294689-5308977	Enterobacteria phage P1	53.23%	NC_005856
	1	23.9Kb	intact	25	528724-552628	Enterobacteria phage P88	53.97%	NC_026014
KP27	2	35.8Kb	intact	45	4521602-4557468	Salmonella phage RE-2010	51.47%	NC_019488
	3	30.7Kb	intact	33	4788378-4819133	Microcystis phage MaMV-DC	54.62%	NC_029002
	4	113.3Kb	intact	115	5078918-5192247	Salmonella phage SSU5	48.98%	NC_018843
	1	74.3Kb	intact	93	557032-631391	Salmonella phage SEN34	53.02%	NC_028699
	2	48.4Kb	intact	62	2255548-2303988	Cronobacter phage ENT47670	51.76%	NC_019927
	3	38.3Kb	intact	37	4312129-4350450	Salmonella phage SEN5	50.06%	NC_028701
KP28	4	82.8Kb	intact	85	4470516-4553348	Enterobacteria phage mEp237	52.78%	NC_019704
	5	29.3Kb	incomplete	29	5280341-5309735	Salmonella phage Fels-1	51.44%	NC_010391
	6	48.5Kb	intact	30	5511889-5560435	Bacteriophage HK022	52.76%	NC_002166
	7	35.1Kb	incomplete	15	5562568-5597762	Pseudomonas phage YMC11/07/P54_PAE_BP	55.44%	NC_030909
KP29	1	37.6Kb	intact	36	4418300-4455922	Salmonella phage SEN4	50.28%	NC_029015
	1	42.7Kb	intact	54	1060592-1103296	Salmonella phage RE-2010	53.01%	NC_019488
	2	58Kb	questionable	64	4880346-4938413	Escherichia phage HK639	54.01%	NC_016158
KP30	3	14.5Kb	incomplete	20	5098179-5112694	Enterobacteria phage P4	46.47%	NC_001609
	4	27.6Kb	intact	39	5355690-5383379	Enterobacteria phage HK140	51.81%	NC_019710
	5	14.1Kb	questionable	18	5484442-5498560	Enterobacteria phage HK544	52.94%	NC_019767
	6	40.3Kb	intact	43	5518810-5559206	Enterobacteria phage mEp235	51.82%	NC_019708
	1	46.5Kb	intact	42	1970872-2017416	Enterobacteria phage Fels-2	44.42%	NC_010463
	2	26.2Kb	incomplete	25	3353187-3379465	Phage Gifsy-1	48.64%	NC_010392
KP31	3	21.4Kb	questionable	21	3376552-3398046	Escherichia phage HK639	52.32%	NC_016158
	4	46.6Kb	intact	32	3690474-3737131	Bacteriophage 186	55.39%	NC_001317
	5	25.5Kb	questionable	28	3930643-3956152	Acinetobacter phage vB_AbaS_TRS1	52.50%	NC_031098

**KP32**	**1**	35.8Kb	intact	44	3224300-3260166	Salmonella phage RE-2010	51.47%	NC_019488
	**2**	37.8Kb	intact	41	4183571-4221425	Salmonella phage SEN5	50.42%	NC_028701
	**3**	121.8Kb	intact	123	4258793-4380657	Salmonella phage SSU5	49.30%	NC_018843
**KQ1**	**1**	49.3Kb	intact	39	1059858-1109185	Enterobacteria phage P88	56.14%	NC_026014
	**2**	80.5Kb	intact	87	4325872-4406402	Klebsiella phage phiKO2	51.84%	NC_005857
	**3**	37.9Kb	intact	43	4434046-4472015	Salmonella phage SEN4	50.23%	NC_029015
	**4**	31.7Kb	questionable	12	5023154-5054908	Staphylococcus phage SPbeta-like	54.74%	NC_029119
**KQ2**	**1**	35.4Kb	intact	44	1015499-1050964	Salmonella phage 118970_sal3	51.30%	NC_031940
	**2**	36.7Kb	intact	29	1052939-1089686	Klebsiella phage phiKO2	53.85%	NC_005857
	**3**	49.7Kb	intact	44	1222156-1271902	Enterobacteria phage P88	56.18%	NC_026014
	**4**	20.3Kb	questionable	10	4611535-4631888	Staphylococcus phage SPbeta-like	52.94%	NC_029119
	**5**	37.9Kb	intact	44	5101140-5139126	Salmonella phage SEN4	50.23%	NC_029015

Supplementary Table S7. PCR primers used for the amplification of the *wzi* and resistance genes.

Target Gene	Primer Name	Sequence 5'-3'	Amplicon Size
wzi[1]	wzi-F	GTGCCGCGAGCGCTTTCTATCTTGGTATTCC	580 bp
	wzi-R	GAGAGCCACTGGTTCCAGAA[C or T]TT[C or G]ACCGC	
bla_{SHV}[2]	SHV-F	GGTTATGCGTTATATTCGCC	865 bp
	SHV-R	TTAGCGTTGCCAGTGCTC	
$bla_{CTX\text{-}M}$[2]	CTX-M-F	ATGTGCAGYACCAGTAARGT	593 bp
	CTX-M-R	TGGGTRAARTARGTSACCAGA	
bla_{IMP}[3]	IMP-F	GGAATAGAGTGGCTTAATTCTC	624 bp
	IMP-R	CCAAACCACTACGTTATC	
bla_{VIM}[4,5]	VIM-F	AGTGGTGAGTATCCGACAG	212 bp
	VIM-R	TCAATCTCCGCGAGAAG	
bla_{KPC}[6]	KPC-F	ATGTCACTGTATCGCCGTC	882 bp
	KPC-R	TTACTGCCCGTTGACGCC	
$bla_{NDM\text{-}1}$[6]	NDM-1-F	TGCATTGATGCTGAGCGGGTG	621 bp
	NDM-1-R	ATCACGATCATGCTGGCCTTG	
$bla_{OXA\text{-}48\text{-}like}$[7]	O48-GDM-F	CCAAGCATTTTTACCCGCATCKACC	389 bp
	O48-GDM-R	GYTTGACCATACGCTGRCTGCG	

Supplementary References:

1. Brisse, S. *et al.* wzi Gene Sequencing, a Rapid Method for Determination of Capsular Type for *Klebsiella Strains*. *J. Clin. Microbiol.* **51,** 4073–4078 (2013).
2. Elumalai, S., Muthu, G., Selvam, R. E. M. & Ramesh, S. Detection of TEM-, SHV- and CTX-M-type β-lactamase production among clinical isolates of *Salmonella* species. *J. Med. Microbiol.* **63,** 962–967 (2014).
3. Li, B. *et al.* Analysis of Drug Resistance Determinants in *Klebsiella pneumoniae* Isolates from a Tertiary-Care Hospital in Beijing, China. *PLOS ONE* **7,** e42280 (2012).
4. Lixandru, B. E. *et al.* Carbapenemase-Producing *Klebsiella pneumoniae* in Romania: A Six-Month Survey. *PLOS ONE* **10,** e0143214 (2015).
5. Pérez-Pérez, F. J. & Hanson, N. D. Detection of Plasmid-Mediated AmpC β-Lactamase Genes in Clinical Isolates by Using Multiplex PCR. *J. Clin. Microbiol.* **40,** 2153–2162 (2002).
6. Wang, D. *et al.* Characterization of the blaKPC-2 and blaKPC-3 genes and the novel blaKPC-15 gene in *Klebsiella pneumoniae*. *J. Med. Microbiol.* **63,** 981–987 (2014).

7. Voets, G. M., Fluit, A. C., Scharringa, J., Cohen Stuart, J. & Leverstein-van Hall, M. A. A set of multiplex PCRs for genotypic detection of extended-spectrum β-lactamases, carbapenemases, plasmid-mediated AmpC β-lactamases and OXA β-lactamases. *Int. J. Antimicrob. Agents* **37,** 356–359 (2011).
